# A combined analytical-chemometric approach for the in vitro determination of polyphenol bioaccessibility by simulated gastrointestinal digestion

**DOI:** 10.1007/s00216-022-03922-x

**Published:** 2022-02-02

**Authors:** Esther Gómez-Mejía, Noelia Rosales-Conrado, María Eugenia León-González, Alejandro Valverde, Yolanda Madrid

**Affiliations:** Analytical Chemistry Department, Faculty of Chemistry, Complutense University of Madrid, 28040 Madrid, Spain

**Keywords:** Tea infusions, Phenolic compounds, In vitro digestion, Bioaccessibility, Liquid chromatography, Multivariate analysis

## Abstract

In this study, an integrated characterisation through polyphenol and caffeine content and antioxidant activity was combined with chemometric analysis to assess the effects of simulated in vitro gastrointestinal digestion on the bioaccessibility of these bioactive compounds from nine different tea infusions. Tea infusions were characterised based on total flavonoids, total polyphenols and antioxidant activity, together with the determination of individual polyphenol content. Fourteen phenolic compounds, including phenolic acids, stilbenes and flavonoids, were selected based on their reported bioactivity and high accessibility, attributed to their low molecular weight. Both polyphenols and caffeine were initially monitored in raw tea infusions and through the different digestion stages (salivary, gastric and duodenal) by capillary high performance liquid chromatography coupled to diode array detection (cHPLC-DAD) and/or HPLC coupled to a triple quadrupole mass analyser (HPLC–MS/MS). Multivariate analysis of the studied bioactives, using principal component analysis and cluster analysis, revealed that the decaffeination process seems to increase the stability and concentration of the compounds evaluated during digestion. The greatest transformations occurred mainly in the gastric and duodenal stages, where low bioactivity indices (IVBA) were shown for resveratrol and caffeic acid (IVBA = 0%). In contrast, the polyphenols gallic acid, chlorogenic acid and quercetin gave rise to their availability in white, green and oolong infusion teas (IVBA > 90%). Furthermore, highly fermented black and pu-erh varieties could be designated as less bioaccessible environments in the duodenum with respect to the tested compounds.

**Supplementary Information:**

The online version contains supplementary material available at 10.1007/s00216-022-03922-x.

## Introduction

Polyphenols are secondary metabolites of plants currently used in pharmaceutical and food industries, with a variety of extensive biological activities [1–3]. Currently, epidemiological studies on the role of polyphenols in nutrition and nutraceuticals are largely based on research on digestion and intestinal absorption, supported by the fact that the most widespread polyphenols in food and beverages are not necessarily the most bioaccessible nor the most bioactive [1].

Tea, obtained by processing of the dried leaves of *Camellia sinensis* plant, is one of the most popular and frequently consumed beverages worldwide, reaching 6.3 million tons [4]. Several tea styles are accessible to the consumer, further differentiated by the type and extent of processing of the leaves: non-fermented (white, yellow and green), partly fermented (oolong), fermented (black tea) and post-fermented (pu-erh) [5], together with their decaffeinated varieties. Emerging experimental and epidemiological studies have revealed that tea consumption exhibits multiple health benefits, attributed to the action of various bioactive phytochemical compounds, particularly polyphenols and purine alkaloids such as caffeine [2, 3].

While many studies have investigated the amount and type of polyphenols present in different teas [6–9], few have reported on the effects of digestive enzymes and pH conditions on the stability of tea polyphenols for human absorption, which may not only influence their bioaccessibility but also their potential activity [10]. Consequently, the studies on bioaccessibility are increasingly considered a feasible first approach to assess the effects of molecular structure and food matrix on the transformations and absorption of polyphenols following food intake [1, 11]. Nevertheless, the scarce research that has addressed the metabolic fate of tea polyphenols once ingested has only focused on the bioaccessibility of catechin and epicatechin derivatives or flavonol glycosides primarily in black, green and white teas [11–14].

Even though catechins and their conjugates are the main constituents in teas, accounting 60 to 80% of total tea polyphenols, it has been stated that it is the metabolites of polyphenols and the smaller phenolic acids that can be absorbed through the intestinal mucosa and, thus, be bioaccessible to reach the target tissues, and exert their effect on health [15]. Therefore, to lay the grounds for further in vivo and epidemiological studies on the role of tea polyphenols in health, it is thus essential to conduct in vitro studies focusing on the behaviour of low molecular weight and simpler polyphenols, which have a more direct biological implication.

Considering that tea origin and processing modify the phenolic profile of tea, and that bioaccessibility is strongly influenced by both the molecular structure and chemical properties of the bioactive compounds, as well as the food matrix [1], information on the digestive behaviour of polyphenols in a broader variety of commercially available teas should be addressed.

On the other hand, the complex interrelationships among the different polyphenols, the food matrix and the digestion steps require recourse to exploratory analyses and unsupervised pattern recognition procedures (e.g. principal component analysis or cluster analysis). Those allow a complete visualisation and understanding of the data set and its associations [8, 16], rather than descriptive statistics as used in existing literature [12, 14].

Therefore, this study was design to investigate to what extent different tea processing conditions not only influence the physico-chemical characteristics (moisture, pH and phenolic profile) and antioxidant activity of tea infusions but also the in vitro bioaccessibility of caffeine and low molecular weight polyphenols, namely, phenolic acids, flavonoids and stilbenes, in different commercial tea varieties.

## Materials and methods

### Solvents and standards

Ethanol (EtOH), acetonitrile (ACN) and methanol (MeOH) of gradient HPLC quality were provided by Scharlab (Barcelona, Spain), and dimethyl sulfoxide (DMSO) was obtained from Sigma-Aldrich (St. Louis, USA). For HPLC–MS/MS analysis, MeOH, water and formic acid (FA) were of Optima™ LC/MS grade (Fisher Scientific, Fair Lawn, NJ, USA). Water was treated in a Milli-Q water purification system (Millipore, Bedford, MA, USA).

Caffeine (≥ 99.0%) and polyphenol standards (95.0–98.0%) were obtained from Sigma-Aldrich (St. Louis, MO, USA) and European Pharmacopoeia. Phenolic stock solutions were prepared in MeOH to a final concentration of 200 mg·L^−1^, and exceptionally, quercetin and hesperidin were dissolved in EtOH-water 80:20 (v/v) and 5% (v/v) DMSO aqueous solution, respectively. Stock solutions were stored in the dark at 4 °C or − 80 °C for no longer than 1 month. Nylon membrane filters (Teknokroma, Barcelona, Spain), with 0.22 µm pore size, were used for mobile phase filtration before LC analysis.

### Tea samples and infusions

*Camellia sinensis* tea leaves from different growing areas have very different production processes, resulting in different varieties, as is illustrated in Figure S1 [5, 17]. The most popular types of tea among Spanish consumers, as well as other lesser known but available varieties, were purchased from the same brand in a local market of Madrid, Spain: white silver needles (Baihao Yinzhen), yellow (Kekecha), green (Sencha), decaffeinated green (Sencha), oolong (Tieguanying), milk oolong (Jin Xuan), black (Ceylon), decaffeinated black (Ceylon) and pu-erh (China).

Tea samples were grounded to obtain a homogeneous fine powder and stored in an opaque and hermetic glass container at 25 °C.

Tea infusions were daily prepared, in triplicate, by pouring 100 mL of Milli-Q water at 95 °C on 0.3000 g of ground tea and brewed for 5 min. After being cooled at 25 °C, the infusions were centrifuged at 8400 rpm for 10 min, and different aliquots were separated for further analysis.

### Moisture and pH determination

The moisture content of teas was obtained according to the Association of Official Analytical Chemists method 20.013 [18]. The pH was measured directly in the cooled tea infusions (pH 555, Teknokroma, Barcelona, Spain).

### Spectrophotometric methods

Total phenolic content (TPC) was performed according to the Folin-Ciocalteu colorimetric method described in a previous work [19]. A volume of 200 μL of tea infusion was mixed in triplicate with 50 μL of Folin-Ciocalteu reagent (Sigma-Aldrich, St. Louis, USA) and 40 μL of Na_2_CO_3_ 7.5% (w/v) (Panreac, Barcelona, Spain). Milli-Q water was added to a final volume of 10 mL. Subsequently, the absorbance was measured at 720 nm (UV–Vis spectrophotometer HP8543, Agilent Technologies). Gallic acid was employed as external standard (0–50 μM, *n* = 5) and the results were expressed as mg of gallic acid equivalents per gram of dried tea (mg GAE∙g^−1^).

The total flavonoid content (TFC) was determined by the aluminium chloride method following the procedure employed by León-González et al. [19]. Tea aliquots of 200 μL were added in triplicate to 2 mL of Milli-Q water, 150 μL of 5% (w/v) NaNO_2_ (Panreac, Barcelona, Spain) and 150 μL of 10% (w/v) AlCl_3_ (Panreac, Barcelona, Spain). After two periods of 5-min reaction, 1 mL of NaOH 1 M (Panreac, Barcelona, Spain) was added, and left to react for other 15 min. Finally, the mixture was diluted to 10 mL. Calibration curves (0–45 μM, *n* = 6) were obtained using quercetin as standard. The absorbance was measured at 415 nm and the results were expressed as mg of quercetin equivalents per gram of dried tea (mg QE∙g^−1^).

Determination of total antioxidant activity (TAA) was based on the molybdenum (VI) reducing power [19], measuring the absorbance of the Mo (V)-phosphate complex at 695 nm. Firstly, an extraction was performed by adding 0.02 g of ground tea to 25 mL of EtOH-H_2_O (80:20 v/v), at 60 °C during 30 min. Following, 100 μL of tea crude extracts were mixed with 1 mL of 0.68 M H_2_SO_4_ (Panreac, Barcelona, Spain), 28 mM Na_3_PO_4_ (Panreac, Barcelona, Spain) and 4 mM (NH_4_)_6_Mo_7_O_24_·4 H_2_O (Panreac, Barcelona, Spain) and incubated at 95 °C for 90 min. Once cooled at 25 °C, Milli-Q water was added to a final volume of 5 mL. Results were obtained through interpolation in the calibration curve (0–15 μM, *n* = 5) using gallic acid as standard, and expressed as gallic acid equivalents per gram of dried tea (mg GAE·g^−1^).

The radical-scavenging activity was evaluated by measuring the absorbance of DPPH radical (1,1-diphenyl–2 picrylhydrazyl) at 515 nm [20]. Five different solutions were prepared in 5 mL, by mixing 2.5 mL of 0.28 mM DPPH (MeOH) and 25–125 µL of tea aliquots. A DPPH control and a blind control (sample infusion aliquot plus MeOH pure solvent) were also prepared. The mixture solutions were left in the dark until the steady-state was attained (20 min for yellow, white and milk oolong teas; 40 min for pu-erh, oolong and green teas; 60 min for decaffeinated black tea; 100 min for decaffeinated green tea and 120 min for black tea). The scavenging ability was calculated in triplicate as DPPH remaining percentage and expressed as milligram of extract per gram of dried tea (mg extract∙g^−1^) at EC_50_ value.

### In vitro* gastrointestinal digestion*

The in vitro bioaccessibility assay was performed according to the procedure described by Tenore et al. [14] slightly modified. Thus, 4 mL of the tea infusion aliquot was mixed with 1 mL of simulated saliva (KCl 89.6 g·L^−1^, KSCN 20 g·L^−1^, NaH_2_PO_4_ 88.8 g·L^−1^, Na_2_SO_4_ 57.0 g·L^−1^, NaCl 175.3 g·L^−1^, NaHCO_3_ 84.7 g·L^−1^, urea 25.0 g·L^−1^, obtained from Panreac, Barcelona, Spain) and 10 mg of α-amylase (Sigma-Aldrich, St. Louis, USA). The pH was adjusted to 6.8 with 0.1 M HCl and incubated at 37 °C for 15 min. Following, 5 mL of 6% (w/v) pepsin (Sigma-Aldrich, St. Louis, USA) dissolved in 0.1 M HCl were added, and the pH was adjusted to 1.8 with 6 M HCl and incubated at 37 °C for 2 h. The duodenal step was initiated by adjusting the pH to 6.8 with NaHCO_3_ saturated aqueous solution. Next, 3 mL of a mixture containing 1.5% (w/v) pancreatin (Sigma-Aldrich, St. Louis, USA) and 0.15% (w/v) bile salts (Sigma-Aldrich, St. Louis, USA) dissolved in 0.15 M NaCl were added. The digested solution finally incubated at 37 °C for 2 h. Solutions were shaken periodically every 10 min while incubating. Gastrointestinal digestion blanks were prepared in parallel. The procedure was performed in triplicate in two/three different days (*n* ≥ 6) and digested samples were freshly analysed to avoid rapid degradation of the analytes.

The bioaccessibility, referred to as the fraction of an ingested compound that is potentially available for absorption during the digestion process, was estimated as bioaccessibility indices (IVBA), i.e. the percentage available for absorption at the duodenal stage, by applying the following Eq. (1):

1 $$IVBA \left(\%\right)= \frac{{[Compound]}_{duodenum}}{{[Compound]}_{initial}}\cdot 100$$

### Determination of phenolic compounds and caffeine

The chromatographic determination of caffeine and polyphenols by cHPLC-DAD was performed on an Agilent system Mod. 1100 Series (Agilent Technologies, Madrid, Spain) with a G1315B diode array detector (500 nL, 10 mm path length). An external stainless-steel loop (10 μL) was placed into a Rheodyne® injection valve and the Agilent Chemstation software package was used for data collection and processing. Chromatographic separation was achieved with a Synergi™ Fusion C18 capillary analytical column (150 mm × 0.3 mm I.D., 4 μm, Phenomenex, Torrance, USA), maintained at room temperature, and using a mobile phase gradient based on a mixture of ACN and 0.1% (v/v) aqueous solution trifluoroacetic acid (TFA, Sigma-Aldrich, St. Louis, USA) at pH 3.2 [19]. Injection volumes were fixed to 10 µL and for the on-column focusing approach, all solutions were prepared in 0.1% (v/v) TFA aqueous solution at pH 3.2, containing 1% (v/v) of ACN. The flow rate was set at 10 µL·min^−1^ and UV detection was performed at 220, 260, 292, 310 and 365 nm. Polyphenols and caffeine were identified by comparing the retention time and their spectral characteristics with those of the standards. Quantitative analyses were performed at the maximum absorbance wavelength (Table S1) using external calibration curves (*n* = 8) obtained for each phenolic pattern (see Table S2). Limits of detection (LODs) and limits of quantification (LOQs) were calculated as 3.3 SD/S and 10 SD/S, respectively. The standard deviation (SD) of the response was established based on the residual SD of a regression curve obtained around LOD concentrations, being the S coefficient of the slope of this calibration curve. Furthermore, the precision was estimated at the following concentrations: 50 and 250 μg·L^−1^ for gallic acid, 20 and 55 μg·L^−1^ for dihydroxybenzoic acid, 125 and 350 μg·L^−1^ for chlorogenic acid, 60 and 350 μg·L^−1^ for caffeine and *p*-coumaric acid, 60 and 175 μg·L^−1^ for *trans*-ferulic acid and rutin, 15 and 60 μg·L^−1^ for resveratrol, and 20 and 50 μg·L^−1^ for quercetin and kaempferol. Intra-day variation (*n* = 3) was evaluated by analysing three standard solutions in the same day and inter-day precision was similarly calculated from three successive days (*N* = 9). Relative standard deviation (RSD, %) was calculated for retention factor (*k*) and peak areas of each analyte (Table S2).

For a more comprehensive identification of bioactive compounds, samples were analysed by HPLC–MS/MS in a LC–MS-8030 triple quadrupole system from Shimadzu Scientific Instruments (Columbia, MD, USA). Data acquisition and processing were performed with the LabSolutions LCMS software (Shimadzu).

HPLC–MS/MS determination was performed on a Synergi™ C18 Fusion-RP 80 Å analytical column (150 mm × 3 mm I.D., 4 µm, Phenomenex, USA), maintained at room temperature, and using a mobile phase gradient based on a mixture of MeOH and 0.2% (v/v) FA aqueous solution. The flow rate was set at 0.50 mL·min^−1^ and the injection volume was fixed at 20 µL [20]. MS injection solutions were prepared in 2.5 mL of MeOH containing 0.2% (v/v) FA and then diluted to 5 mL with LC/MS grade water.

Mass spectral detection was carried out by means of multiple reaction monitoring (MRM) mode with a 100-ms-dwell time. The ESI source worked in negative ionisation mode for phenolic compounds (M − H^−^) and in positive ionisation mode for caffeine (M + H^+^). Three selective transitions were registered for each parent compound, and the most abundant one was used for quantitative purposes (Table S1). Linearity of the HPLC–MS/MS was evaluated at five concentration levels by means of external calibration curves. The method performance was assessed as previously described in terms of limit of detection, limit of quantification and reproducibility (Table S2). The latter was calculated for peak areas at the following concentrations: 28 μg·L^−1^ for quercetin and myricetin; 35 μg·L^−1^ for *trans*-ferulic acid and kaempferol; 45 μg·L^−1^ for resveratrol, hesperidin, caffeic acid, *p*-coumaric acid and dihydroxybenzoic acid; 54 μg·L^−1^ for chlorogenic acid; 60 μg·L^−1^ for rutin and naringin; 70 μg·L^−1^ for caffeine and catechin; and 80 μg·L^−1^ for gallic acid.

### Statistical analysis

Spectrophotometric and chromatographic data were evaluated by one-way analysis of variance (ANOVA), least significant difference (LSD), cluster analysis (CA) and principal component analysis (PCA). The software package used was *Statgraphics Centurion 19* (Statgraphics Technologies, Inc., Rockville, MD, USA).

## Results and discussion

### Characterisation of tea infusions

#### Physico-chemical, total contents and antioxidant characteristics

Tea infusions were characterised in terms of pH, TPC, TFC and antioxidant activities (TAA and DPPH). Moreover, the moisture content of the tea leaves used for tea infusion preparation was determined.

The moisture content in the tea leaves and the pH of the infusions affect the quality (aroma, colour and flavour) and/or shelf life of the commercialised product. In addition, the processing of tea leaves can modify both properties, leading to changes in many phenomena, such as protein properties, enzymatic activities, growth of microorganisms or chemical reactions, which may also alter the phytochemical profile of tea infusions. Thus, knowledge of pH and moisture is a primary parameter for performing tea characterisation and studying tea processing [21, 22].

**Fig. 1:**
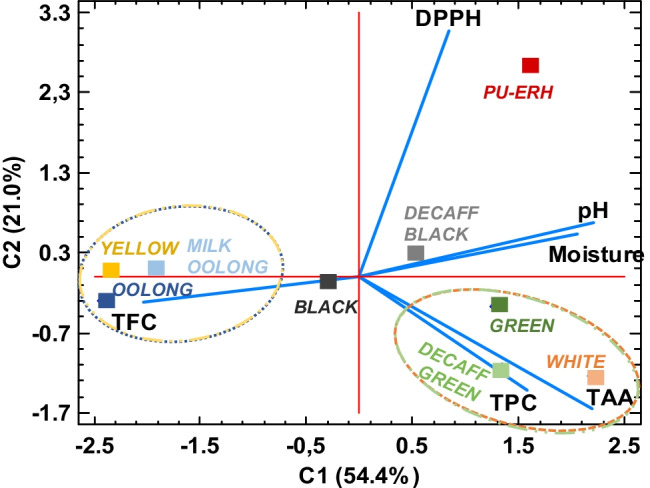
2D PCA bi-plot of nine tea infusions (scores, mean values *n* = 3) based on the total polyphenol content (TPC), total flavonoid content (TFC), total antioxidant activity (TAA), DPPH radical-scavenging ability, pH and moisture. Ellipses show clustering of the samples

On the other hand, spectrophotometric determinations (TPC, TFC, TAA and DPPH) are widely applied in the analysis of phenolic compounds in food and beverages [5, 23, 24], as they provide rapid and useful information on the variability of phenolic composition [19]. Table 1 shows the experimental mean values obtained for the aforementioned assays. These obtained results were subjected to a PCA analysis to visually assess the associations between tea types and determinations. Figure 1 shows the two-dimensional representation of nine tea infusion studies, which explained 75.4% of the data set, and presents a direct correlation between moisture and pH, reversed to TFC. Yellow, oolong and milk oolong varieties had the lowest moisture and pH values, whereas the TFC ones were the highest. Thus, the sweltering and partial oxidation conducted in the manufacture of yellow and oolong teas, respectively, play a key role in decreasing the moisture and enhancing the content of acidic phytochemicals, as well as total flavonoids. Similar results have been reported by Zhang et al. [9], founding higher flavonoid contents, namely, catechin derivatives such as epigallocatechin or gallocatechin gallate, in yellow and oolong tea compared to other tea varieties.

**Table 1:** Comparison of moisture, pH, TPC, TFC and antioxidant activities (using Mo test reduction and DPPH radical-scavenging ability) of tea infusions. Data are expressed as mean ± standard deviation (*n* = 3)

Tea variety	Moisture (%)	pH	TPC (mg GAE·g^−1^ DW)	TFC (mg QE·g^−1^ DW)	TAA (mg GAE·g^−1^ DW)	DPPH assay (mg extract g^−1^ sample DW)
White	7.7 ± 0.1^b^	5.5 ± 0.2^ab^	30 ± 6^a^	38 ± 16^a^	689 ± 90^a^	3.3 ± 0.3^a^
Yellow	5.4 ± 0.1^e^	4.9 ± 0.4^de^	12 ± 3^bc^	283 ± 3^b^	47 ± 2^b^	6.0 ± 0.2^b^
Green	7.5 ± 0.2^b^	5.4 ± 0.2^ab^	31 ± 6^a^	105 ± 5^c^	473 ± 32^c^	6.4 ± 0.9^b^
Decaffeinated green	6.5 ± 0.2^c^	5.3 ± 0.2^bc^	66 ± 3^d^	103 ± 4^c^	441 ± 38^c^	6.5 ± 0.3^b^
Oolong	4.6 ± 0.1^f^	4.7 ± 0.2^de^	8 ± 1^b^	153 ± 15^d^	30 ± 6^b^	4.0 ± 0.1^ac^
Milk oolong	5.6 ± 0.1^d^	4.7 ± 0.4^e^	11 ± 1^bc^	142 ± 7^d^	32 ± 5^b^	5.8 ± 0.9^b^
Black	6.6 ± 0.2^c^	5.0 ± 0.2^ cd^	5 ± 4^b^	61 ± 14^e^	228 ± 75^de^	4.3 ± 0.4^c^
Decaffeinated black	8.91 ± 0.01^a^	4.8 ± 0.2^de^	29 ± 5^a^	121 ± 10^c^	299 ± 63^d^	8.5 ± 0.6^d^
Pu-erh	7.53 ± 0.03^b^	5.6 ± 0.2^a^	18 ± 3^c^	51 ± 11^ae^	183 ± 23^e^	15.9 ± 0.4^e^

Mean values with different letters in the same column indicate significant differences at *p*-values < 0.05, according to ANOVA and Fisher LSD tests. *TPC*, total polyphenol content; *GAE*, gallic acid equivalents; *TFC*, total flavonoid content; *QE*, quercetin equivalents; *TAA*, total antioxidant activity; *DW*, dry weight

In contrast, post-fermentation of pu-erh tea resulted in a slight basification of the infusion and a higher moisture content, which favours the enzymatic activity required for developing its desirable flavour. This enzymatic oxidation is accompanied by a poor radical-scavenging activity (DPPH assay), which may be attributed to the loss of simple tea flavonoids, transformed into complex theaflavins and thearubigins [5, 25].

Alternatively, a positive correlation was found between TPC and TAA (Fig. 1), indicating that teas enriched with phenolic compounds, and in particularly with monomeric polyphenols such as catechin, are more likely to exhibit a better antioxidant activity through reducing capacity, as also described elsewhere [14, 23, 26]*.* The low oxidation level of white and green tea promotes a larger content of these monomeric polyphenols, and as a result, the highest of TPC and TAA values were observed for white, green and decaffeinated green tea. However, both green and black decaffeinated teas showed higher TPC values than their caffeinated analogues. Since caffeine has been found to be a prooxidant, whose presence during food processing may induce oxidation of some phenolic compounds [10] and thus reducing their TPC values. Overall, the spectrophotometric profile, pH and moisture seemed to be further altered in fermented black tea than in unfermented green tea. This fact was also noted by Carloni et al. [23], who found no significant differences for the TPC, TFC and antioxidant activities in green and low-caffeine green tea.

#### Individual determination of polyphenols and caffeine

Although spectrophotometric methods have been widely used for the estimation of total phenolic and flavonoid content, as well as the antioxidant activity, their lack of selectivity can be misleading and lead to overestimated results [27]. For this reason, chromatographic methods are required to assess the effect of processing on the in vitro bioaccessibility and the individual phenolic content of different tea varieties. Thus, cHPLC-DAD and HPLC–MS/MS were employed to determine the individual concentration of polyphenols and caffeine in the tea infusions studied. The use of HPLC–MS/MS enabled the quantification of some compounds not possibly quantifiable in some digested samples by cHPLC-DAD, due to overlap with some endogenous matrix material. The HPLC–MS/MS analysis confirmed the presence of caffeine, gallic, dihydroxybenzoic, chlorogenic and *p*-coumaric acid, rutin, narangin and quercetin, at concentration levels consistent with those obtained by the cHPLC-DAD analysis. As a novelty, catechin, caffeic acid, hesperidin and myricetin were identified and quantified by HPLC–MS/MS, when possible. However, ferulic acid, resveratrol and kaempferol were not detected at the LOD of the HPLC–MS/MS method. The low sensitivity of HPLC–MS/MS for resveratrol, kaempferol and ferulic, compared to that of the cHPLC-DAD method, could be attributed to the negative ionisation mode employed, which reportedly reduces sensitivity and the impairment of ionisation triggered by the complex matrix or the improvement of the LOD when capillary LC columns and on-column focusing techniques are employed [28]. Table 2 shows the polyphenolic and caffeine contents in non-digested and digested tea infusions. As an illustration, Figure S2 and Figure S3 show the HPLC–DAD chromatograms and HPLC–MS/MS spectra of green tea infusion and the respective digested extracts.

**Table 2:** Polyphenolic and caffeine contents determined by HPLC in non-digested (raw) and digested tea infusions (mean ± SD, *n* ≥ 6)

	**Concentration (mg·g**^**−1**^** DW tea)**							
	***White***	***W salivar***	***W gastric***	***W duodenal***	***Yellow***	***Y salivar***	***Y gastric***	***Y duodenal***
**Gallic acid**	13.4 ± 0.9^a^	14.2 ± 0.9^a^	11.4 ± 0.9^b^	3.75 ± 0.4^c^	4.7 ± 0.5^a^	3.4 ± 0.4^b^	0.59 ± 0.01^c^	0.89 ± 0.01^d^
**DHB**	0.070 ± 0.002^a**^	nd	nd	nd	0.39 ± 0.04^a^	0.3 ± 0.2^a*^	0.59 ± 0.01^b^	0.267 ± 0.004^a**^
**Caffeine**	64 ± 5^a^	64 ± 5^a^	60 ± 6^a^	59 ± 5^a^	42 ± 3^a^	38 ± 7^a^	27 ± 5^b^	30 ± 5^b^
**Caffeic**	nd	nd	nd	nd	nd	nd	nd	nd
**Catechin**	2.9 ± 0.1^a^	3.0 ± 0.6^a^	3.0 ± 0.6^a^	1.33 ± 0.01^b^	nd	nd	nd	nd
**Chlorogenic**	0.61 ± 0.02^ab^	0.69 ± 0.06^b^	0.60 ± 0.03^ab^	0.55 ± 0.01^a^	0.5 ± 0.2^a^	0.63 ± 0.07^b^	0.20 ± 0.08^c**^	0.6 ± 0.2^ab^
***p*-Coumaric**	1.8 ± 0.1^a^	1.8 ± 0.2^a^	1.4 ± 0.1^b^	1.08 ± 0.05^c^	nd	nd	nd	nd
**Ferulic**	0.91 ± 0.08^a^	0.9 ± 0.1^a^	1.3 ± 0.1^b^	1.19 ± 0.08^c^	nd	nd	nd	nd
**Rutin**	63 ± 6^a^	62 ± 7^a^	29 ± 3^b^	23 ± 2^c^	3.7 ± 0.3^a^	3.5 ± 0.6^a^	2.8 ± 0.2^b^	2.4 ± 0.5^b^
**Narangin**	0.16 ± 0.01^a**^	nd	nd	nd	0.16 ± 0.05^a**^	nd	nd	nd
**Hesperidin**	0.234 ± 0.008^a*^	0.29 ± 0.01^a*^	0.78 ± 0.03^b*^	1.8 ± 0.1^c^	1.18 ± 0.09^a^	1.19 ± 0.05^a^	0.593 ± 0.009^b*^	0.267 ± 0.004^c**^
**Myrecetin**	0.18 ± 0.03^a**^	nd	nd	0.132 ± 0.005^b**^	nd	nd	nd	nd
**Resveratrol**	nd	nd	nd	nd	0.107 ± 0.002^a**^	0.134 ± 0.002^b*^	nd	nd
**Quercetin**	0.117 ± 0.004^a*^	nd	0.39 ± 0.01^b*^	nd	0.69 ± 0.07^a^	0.34 ± 0.01^b^	nd	nd
**Kaempferol**	nd	nd	1.56 ± 0.05^a^	nd	0.55 ± 0.08^a^	0.5 ± 0.1^a^	0.273 ± 0.004^b^	nd
	***Green***	***G salivar***	***G gastric***	***G duodenal***	***Decaff. green***	***DG salivar***	***DG gastric***	***DG duodenal***
**Gallic acid**	1.7 ± 0.1^a^	1.7 ± 0.2^a^	2.46 ± 0.04^b^	2.9 ± 0.2^c^	0.8 ± 0.1^a^	0.8 ± 0.1^a^	1.36 ± 0.01^b^	0.9 ± 0.2^a^
**DHB**	0.240 ± 0.003^a^	0.307 ± 0.005^b^	nd	nd	nd	nd	nd	nd
**Caffeine**	31 ± 2^a^	30 ± 3^a^	26 ± 1^b^	24 ± 3^b^	6.1 ± 0.7^a^	5.8 ± 0.4^a^	2.8 ± 0.1^b^	3.2 ± 0.4^b^
**Caffeic**	0.074 ± 0.001^a**^	nd	nd	nd	nd	nd	nd	nd
**Catechin**	0.74 ± 0.03^a^	0.94 ± 0.06^ab^	1.1 ± 0.2^b**^	nd	1.4 ± 0.2^a^	1.5 ± 0.1^a^	1.20 ± 0.03^a*^	0.40 ± 0.01^b**^
**Chlorogenic**	0.17 ± 0.02^a*^	0.15 ± 0.02^a*^	0.24 ± 0.07^a**^	nd	0.110 ± 0.001^a**^	0.116 ± 0.001^a**^	0.19 ± 0.02^b**^	0.17 ± 0.01^b**^
***p*-Coumaric**	0.78 ± 0.04^a^	0.69 ± 0.06^b^	1.4 ± 0.1^c^	1.2 ± 0.1^d^	0.94 ± 0.03^a^	0.92 ± 0.05^a^	1.6 ± 0.1^b^	1.71 ± 0.09^ab^
**Ferulic**	1.0 ± 0.1^a^	0.98 ± 0.07^ab^	1.0 ± 0.1^ab^	0.9 ± 0.1^b^	1.30 ± 0.07^a^	1.28 ± 0.08^a^	1.4 ± 0.1^b^	1.3 ± 0.1^ab^
**Rutin**	27 ± 2^a^	25 ± 2^b^	21 ± 1^c^	18 ± 2^d^	35 ± 1^a^	33 ± 2^a^	31 ± 2^b^	33 ± 3^ab^
**Narangin**	0.090 ± 0.006^a**^	nd	nd	nd	0.39 ± 0.02^a*^	nd	nd	nd
**Hesperidin**	0.246 ± 0.004^a**^	0.307 ± 0.005^a**^	0.78 ± 0.07^b**^	nd	0.239 ± 0.005^a*^	0.299 ± 0.006^b*^	0.80 ± 0.02^c*^	nd
**Myricetin**	0.123 ± 0.002^a**^	nd	nd	nd	nd	nd	nd	nd
**Resveratrol**	0.25 ± 0.03^a^	0.20 ± 0.02^b^	nd	nd	0.30 ± 0.02^a^	0.30 ± 0.03^a^	0.21 ± 0.02^b^	nd
**Quercetin**	0.123 ± 0.002^a**^	nd	0.45 ± 0.07^b**^	nd	0.12 ± 0.03^a^	0.15 ± 0.03^a^	0.5 ± 0.1^b^	nd
**Kaempferol**	nd	nd	nd	nd	nd	nd	nd	nd
	**Concentration (mg·g**^**−1**^** DW tea)**							
	***Oolong***	***O salivar***	***O gastric***	***O duodenal***	***Milk oolong***	***MO salivar***	***MO gastric***	***MO duodenal***
**Gallic acid**	0.9 ± 0.1^a^	0.9 ± 0.1^a^	0.58 ± 0.01^b*^	0.245 ± 0.003^c**^	0.98 ± 0.07^a^	0.7 ± 0.1^b^	0.60 ± 0.01^c^	0.270 ± 0.003^d**^
**DHB**	0.4 ± 0.1^a^	0.218 ± 0.003^bc*^	0.175 ± 0.002^c**^	0.262 ± 0.003^b**^	0.180 ± 0.002^a**^	0.225 ± 0.002^b*^	0.180 ± 0.002^a**^	0.269 ± 0.003^c**^
**Caffeine**	28 ± 2^a^	27 ± 2^a^	20 ± 3^b^	16 ± 4^c^	24 ± 1^a^	25 ± 1^a^	16 ± 2^b^	4 ± 1^c^
**Caffeic**	nd	nd	nd	nd	nd	nd	nd	nd
**Catechin**	nd	nd	nd	nd	nd	nd	nd	nd
**Chlorogenic**	0.210 ± 0.003^a**^	0.249 ± 0.005^b**^	nd	nd	0.158 ± 0.002^a**^	0.175 ± 0.002^b**^	nd	nd
***p*-Coumaric**	0.118 ± 0.001^a^	nd	nd	nd	nd	nd	nd	nd
**Ferulic**	nd	nd	nd	nd	nd	nd	nd	nd
**Rutin**	2.5 ± 0.4^a^	1.9 ± 0.5^b^	2.0 ± 0.6^ab^	1.87 ± 0.02^b^	2.9 ± 0.2^a^	1.0 ± 0.2^b^	1.9 ± 0.2^c^	1.92 ± 0.02^c^
**Narangin**	nd	nd	nd	nd	nd	nd	nd	nd
**Hesperidin**	0.7 ± 0.1^a^	0.6 ± 0.1^ab^	0.58 ± 0.01^b*^	0.262 ± 0.003^c**^	0.7 ± 0.1^a^	0.49 ± 0.06^b^	0.60 ± 0.01^c*^	0.269 ± 0.003^d**^
**Myricetin**	nd	nd	nd	nd	nd	nd	nd	nd
**Resveratrol**	0.19 ± 0.01^a^	0.23 ± 0.01^b^	0.186 ± 0.002^a**^	nd	0.112 ± 0.004^a^	0.11 ± 0.01^a*^	nd	nd
**Quercetin**	0.70 ± 0.05^a^	0.65 ± 0.09^a^	1.0 ± 0.1^b*^	1.3 ± 0.1^c**^	0.55 ± 0.01^a^	0.55 ± 0.07^a^	1.0 ± 0.1^b*^	0.92 ± 0.09^b**^
**Kaempferol**	nd	nd	nd	nd	0.278 ± 0.003^a*^	0.103 ± 0.001^b^	nd	nd
	***Black***	***B salivar***	***B gastric***	***B duodenal***	***Decaff. black***	***DB salivar***	***DB gastric***	***DB duodenal***
**Gallic acid**	4.9 ± 0.2^a^	2.7 ± 0.1^b^	5.2 ± 0.3^a^	2.66 ± 0.01^b^	6.2 ± 0.2^a^	6.2 ± 0.3^a^	6.4 ± 0.7^a^	2.7 ± 0.2^b^
**DHB**	0.236 ± 0.001^a*^	0.16 ± 0.02^b*^	nd	nd	0.11 ± 0.01^a**^	0.10 ± 0.01^a**^	nd	nd
**Caffeine**	36 ± 2^a^	37 ± 1^a^	32 ± 3^a^	29 ± 2^a^	1.0 ± 0.4^a^	0.95 ± 0.05^a^	0.85 ± 0.01^ab^	0.36 ± 0.02^b^
**Caffeic**	0.212 ± 0.01^a*^	0.118 ± 0.001^b*^	nd	nd	0.231 ± 0.01^a*^	0.14 ± 0.01^b**^	nd	nd
**Catechin**	0.41 ± 0.03^a^	nd	nd	nd	0.5 ± 0.2^a^	nd	nd	nd
**Chlorogenic**	0.31 ± 0.02^a^	0.26 ± 0.01^a*^	0.39 ± 0.02^b*^	0.31 ± 0.04^a**^	0.45 ± 0.03^a^	0.39 ± 0.02^b^	0.569 ± 0.001^c^	0.42 ± 0.02^ab**^
***p*-Coumaric**	0.61 ± 0.03^a^	0.54 ± 0.05^b^	0.63 ± 0.03^a^	0.66 ± 0.08^a^	0.77 ± 0.04^a^	0.66 ± 0.05^b^	0.62 ± 0.06^bc^	0.60 ± 0.05^c^
**Ferulic**	1.10 ± 0.07^a^	0.87 ± 0.06^b^	0.76 ± 0.09^c^	0.81 ± 0.07^bc^	0.61 ± 0.05^a^	0.45 ± 0.04^b^	0.47 ± 0.07^b^	0.27 ± 0.05^c^
**Rutin**	5.7 ± 0.5^a^	4.0 ± 0.5^b^	2.9 ± 0.6^c^	2.1 ± 0.9^d^	11.4 ± 0.5^a^	9 ± 1^b^	8 ± 1^c^	6.2 ± 0.3^d^
**Narangin**	nd	nd	nd	nd	nd	nd	nd	nd
**Hesperidin**	0.236 ± 0.001^a*^	0.295 ± 0.001^b*^	0.590 ± 0.002^c*^	nd	0.24 ± 0.01^a^	nd	nd	nd
**Myricetin**	nd	nd	nd	nd	nd	nd	nd	nd
**Resveratrol**	0.19 ± 0.02^a^	0.16 ± 0.01^b^	nd	nd	0.12 ± 0.01^a^	0.07 ± 0.01^b**^	nd	nd
**Quercetin**	0.29 ± 0.02^a^	0.27 ± 0.02^b^	nd	nd	0.36 ± 0.03^a^	0.342 ± 0.01^b^	nd	nd
**Kaempferol**	nd	nd	nd	nd	nd	nd	nd	nd
**Concentration (mg·g**^**−1**^** DW tea)**								
	***Pu-erh***	***P salivar***	***P gastric***	***P duodenal***				
**Gallic acid**	2.3 ± 0.3^a^	1.7 ± 0.2^b^	0.60 ± 0.01^c*^	0.271 ± 0.003^d**^				
**DHB**	0.45 ± 0.01^a^	0.226 ± 0.002^b*^	0.60 ± 0.01^c*^	0.271 ± 0.003^d**^				
**Caffeine**	31 ± 5^a^	35 ± 2^a^	33 ± 5^a^	26 ± 3^b^				
**Caffeic**	0.97 ± 0.06^a^	0.65 ± 0.02^b^	0.58 ± 0.06	nd				
**Catechin**	nd	nd	nd	nd				
**Chlorogenic**	0.217 ± 0.002^a**^	0.271 ± 0.003^b**^	nd	nd				
***p*-Coumaric**	0.15 ± 0.01^a**^	nd	nd	nd				
**Ferulic**	0.063 ± 0.004^a**^	nd	nd	nd				
**Rutin**	1.3 ± 0.2^a^	0.39 ± 0.03^b^	1.29 ± 0.01^a*^	0.632 ± 0.01^c**^				
**Narangin**	nd	nd	nd	nd				
**Hesperidin**	0.5 ± 0.1^a^	0.46 ± 0.04^a*^	nd	nd				
**Myricetin**	0.21 ± 0.03^a*^	nd	nd	nd				
**Resveratrol**	0.19 ± 0.02^a^	0.08 ± 0.01^b**^	nd	nd				
**Quercetin**	0.37 ± 0.04^a^	0.39 ± 0.03^a^	0.379 ± 0.004^a*^	nd				
**Kaempferol**	0.279 ± 0.03^a^	nd	nd	nd				

*W*, white tea; *Y*, yellow tea; *G*, green tea; *DG*, decaff. green tea; *O*, oolong tea; *MO*, milk oolong tea; *B*, black tea; *DB*, decaff. black tea; *P*, pu-erh tea; *nd*, nondetected. For each tea, mean values with different superscript for the same analyte reveal significant differences at *p-*values < 0.05, according to ANOVA and Fisher LSD test

^*^Value obtained through the LOQ, ^**^value obtained through the LOD

**Fig. 2:**
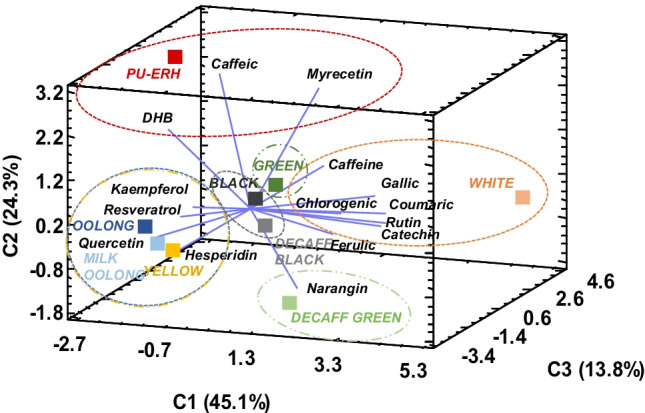
Three-dimensional PCA plot of nine tea infusions (scores, mean values *n* ≥ 6) according to the individual polyphenol content and caffeine determined by cHPLC-DAD and/or HPLC–MS/MS. Ellipses show clustering of the samples

The individual pattern of the studied standard polyphenols and caffeine of the raw tea infusions was assessed according to a PCA, in which three principal components explained 83.2% of the total data variance. The data set was plotted to produce a three-dimensional graph, as shown in Fig. 2, in which the tea varieties were clustered into different groups, denoting a meaningful influence of processing on the amount and distribution of the bioactive compounds in teas. As can be seen, white, green and green decaffeinated infusions were characterised by the highest levels of *p-*coumaric acid, gallic acid, catechin, narangin, rutin and caffeine. These values are the result of the non-fermented nature of the teas aforementioned, where conjugation of simple phenolic acids and flavonoids is not favoured and there is a more active metabolism of caffeine and a lower sublimation [5, 7, 14]. Noteworthy, yellow tea showed a rather distinctive phenolic profile compared to the other non-fermented varieties (white and green tea), with low amounts of rutin, *p-*coumaric acid and catechin. This might be explained by the unique manufacturing process of yellow variety by sweltering in a closed humid container [8], which makes it rich in other flavonoids such as quercetin, hesperidin and kaempferol.

On the other hand, the oxidation initiated in oolong teas, triggered by polyphenol oxidase and peroxidase after the rolling step, facilitates the degradation and polymerisation of the simple tea polyphenols [29], resulting in a complex mixture of oxidative products (theaflavin, thearubigen and theabrownins) and native low molecular weight molecules, such as hesperidin and quercetin. Additionally, the blue-making oxidation accounts for the decrease in catechin and gallic acid, along with caffeine (see also Table 2). Nonetheless, this trend seemed to be reversed in black teas, bringing them closer to unfermented green tea, where the increase in catechin and gallic acid is suggested to be associated by the oxidative degallation of phenolic esters through leaf crushing in the rolling step, as stated elsewhere [5, 30]. Pu-erh tea undergoes a post-fermentation following the enzymatic activity of microorganisms such as *Aspergillus* sp. [8]. As a result, extensive loss of catechins, rutin, ferulic acid, *p-*coumaric acid and narangin was observed, in favour of the formation of both, complex phenolic polymers and their smaller hydrolytic metabolites [8, 15], such as caffeic acid, dihydroxybenzoic acid and myricetin (Fig. 2). In fact, myricetin was mainly determined in fermented teas (black tea, decaffeinated black tea and pu-erh tea), as it has been claimed to be scarce in non-fermented teas [15], appearing in low concentrations only in white and green tea (Table 2).

In addition, a greater impact of decaffeination on the content and distribution of the simple polyphenols studied was especially observed in green tea compared to black one, as the former undergoes a subsequent oxidation process that could counter the effect of decaffeination. This result was not observed in the spectrophotometric characterisation shown in Fig. 1, demonstrating the need to employ chromatographic methods to accurately analyse the effect of processing on the phenolic profile of teas.

#### Simulated gastrointestinal digestion and bioaccessibility study

The release of simple low molecular weight tea polyphenols and caffeine was monitored during in vitro digestion, and it was compared with the polyphenolic content and distribution natively present in the tested teas, so as to subsequently estimate their bioaccessibility indices (IVBA) which are included in Table 3.

**Table 3:** Bioaccessibility indices (IVBA) of polyphenols and caffeine calculated for digested tea infusions (mean ± SD, *n* ≥ 6)

	**IVBA (%)**								
	***White***	***Yellow***	***Green***	***Decaff. green***	***Oolong***	***Milk oolong***	***Black***	***Decaff. black***	***Pu-erh***
**Gallic acid**	32 ± 2^d^	19 ± 2^ef^	174 ± 11^a^	109 ± 16^b^	27 ± 3^de^	28 ± 2^de^	55 ± 2^c^	43 ± 2^c^	12 ± 1^f^
**DHB**	0	70 ± 6^b^	0	-	66 ± 9^c^	150 ± 9^a^	0	0	60 ± 2^d^
**Caffeine**	92 ± 7^a^	71 ± 11^c^	75 ± 6^c^	53 ± 6^d^	54 ± 14^d^	18 ± 4^f^	79 ± 5^bc^	38 ± 5^e^	85 ± 15^ab^
**Caffeic**	-	-	0	-	-	-	0	0	0
**Catechin**	46 ± 1^a^	-	0	30 ± 4^b^	-	-	0	0	-
**Chlorogenic**	90 ± 5^c^	98 ± 10^b^	0	154 ± 6^a^	0	0	99 ± 8^b^	93 ± 1^bc^	0
***p*-Coumaric**	59 ± 2^e^	-	153 ± 11^b^	181 ± 10^a^	0	-	107 ± 12^c^	78 ± 7^d^	0
**Ferulic**	131 ± 14^a^	-	89 ± 8^c^	102 ± 10^b^	-	-	74 ± 10^d^	45 ± 9^e^	0
**Rutin**	37 ± 6^f^	67 ± 12^bc^	65 ± 6^c^	95 ± 6^a^	75 ± 13^b^	67 ± 5^bc^	36 ± 14^f^	54 ± 3^d^	51 ± 7^d^
**Narangin**	0	0	0	0	-	-	-	-	-
**Hesperidin**	769 ± 78^a^	23 ± 2^c^	0	0	39 ± 9^b^	39 ± 7^b^	0	0	0
**Myricetin**	78 ± 14	-	0	-	-	-	-	-	0
**Resveratrol**	-	0	0	0	0	0	0	0	0
**Quercetin**	0	0	0	0	180 ± 12^a^	167 ± 17^b^	0	0	0
**Kaempferol**	-	0	-	-	-	0	-	-	0

(-) The compound was not determined in the initial infusion and the IVBA was not calculated. Mean values with different superscript for the same analyte indicate significant differences at *p-*values < 0.05, according to ANOVA and Fisher LSD test

**Fig. 3:**
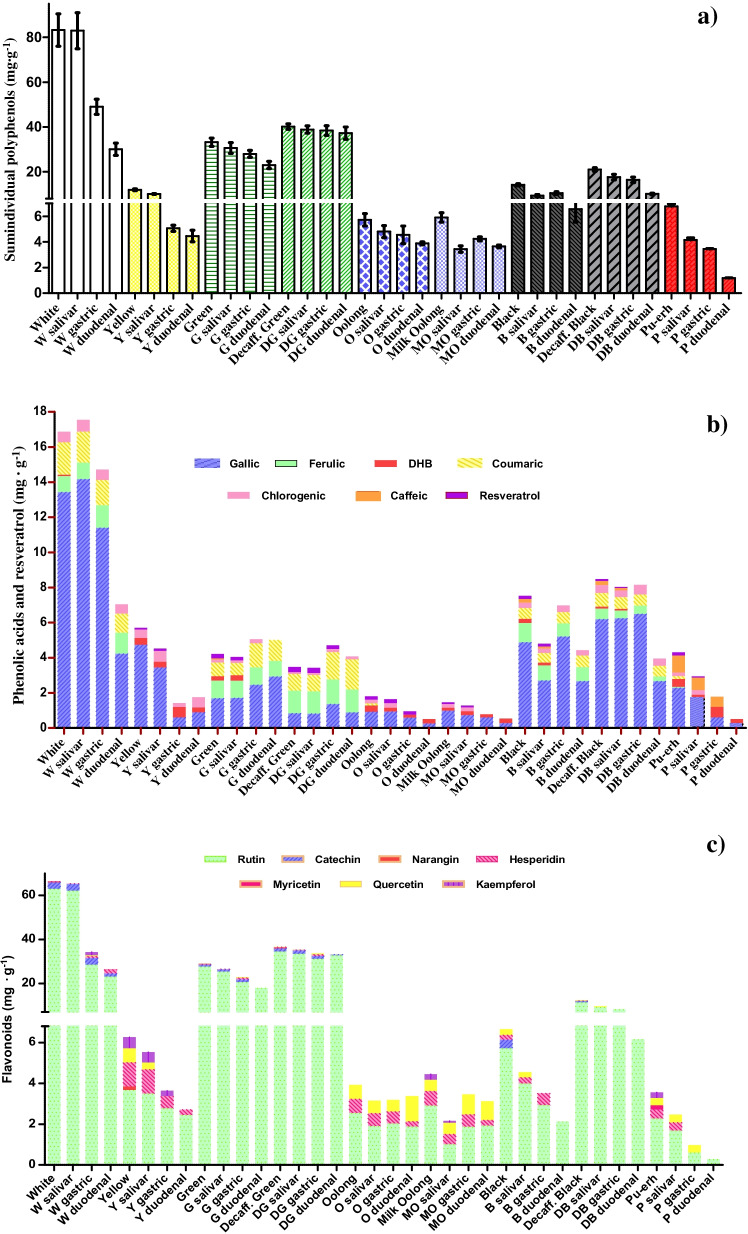
Concentrations of bioactive polyphenols determined by cHPLC-DAD and HPLC–MS/MS at each digestion step for the different tea infusions evaluated: **a** sum of individual polyphenols, **b** phenolic acids and resveratrol, **c** flavonoids

Figure 3 shows the variation of the individual contents of the bioactive polyphenols determined at each stage of digestion by cHPLC-DAD and HPLC–MS/MS. According to Fig. 3a, the highest total individual phenolic concentration was observed in the non-fermented white (83–30 mg·g^−1^) and green teas (40–23 mg·g^−1^), throughout the whole digestion process, agreeing to their lower grade of polymerisation. Once again, it was noted that decaffeinated varieties stood out for showing higher amounts of total individual polyphenols (40–37 mg·g^−1^ for decaffeinated green and 21–10 mg·g^−1^ for decaffeinated black) compared to caffeinated green (33–23 mg·g^−1^) and black tea (14–7 mg·g^−1^) digestion steps, probably due to the prooxidant activity of caffeine and the deactivation of polyphenol oxidase [10, 31]. Moreover, the absence of caffeine and the lower fermentation manufacture of green tea seem to ease the overall loss of polyphenols during the digestion process.

**Fig. 4:**
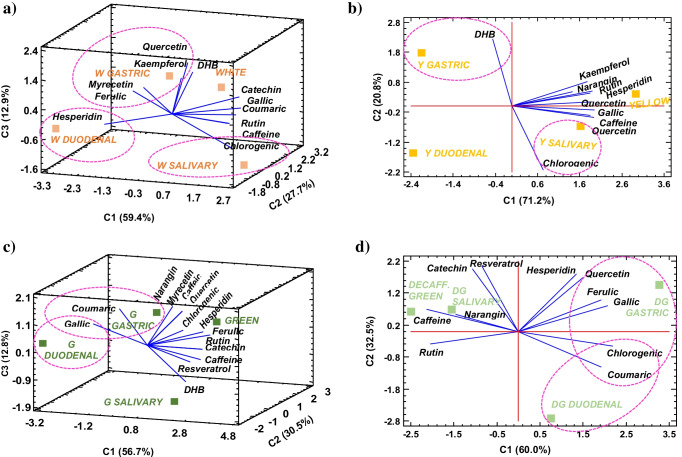

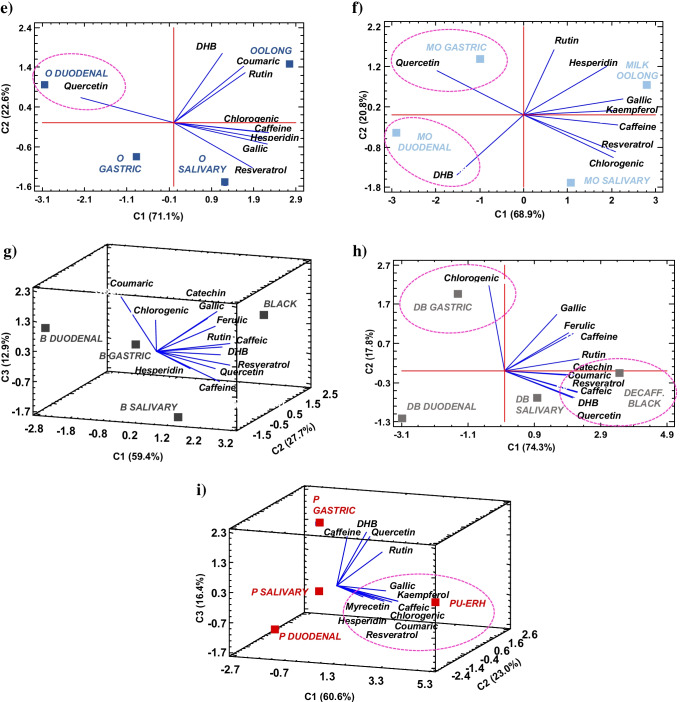
2D and 3D PCA graphics of the in vitro digestion process in each tea infusion variety (scores, mean values *n* ≥ 6): **a** white, **b** yellow, **c** green, **d** green decaffeinated, **e** oolong, **f** milk oolong, **g** black, **h** black decaffeinated and **i** pu-erh. The ellipses show the association of the analytes (loadings) at the digestion stages

To summarise and enlighten the differences in polyphenol and caffeine digestion for the different tea varieties studied, plus to establish the relationship between the bioactive compounds evaluated, a multivariate PCA was conducted for each tea type (Fig. 4). This analysis allowed variations in phenolic stability to be observed graphically with minimal loss of information and correlated with tea manufacture and phenolic composition.

#### Tea phenolic acids and resveratrol

Chlorogenic acid was quantified in all tea infusions evaluated showing high stability in digestive environments for both unfermented and fully fermented teas (0.11–0.69 mg·g^−1^). For white and yellow teas, an increase was observed in the salivary phase (Fig. 4a and b), where a low stability of chlorogenic derivatives triggered by a mild alkaline pH appeared to enhance its content. Also, an increase in chlorogenic concentration was observed in the gastric phase of regular and decaffeinated green and black teas (Fig. 4c, d, g and h), possibly caused by acid hydrolysis of other matrix components bound to the chlorogenic moiety in the stomach [32]. Then again, the mild alkaline pH found in the intestinal phase, as well as the formation of pancreatin-chlorogenic complex through Van der Waal interactions and hydrogen bonds, might have caused a small decrease in the concentration of chlorogenic acid, as it has been observed by other authors [10, 33]. Even though IVBA higher than 90% were reported. Therefore, the low degradation of chlorogenic acid suggests that the attributed health benefits, such as anti-anxiety effects, after intraperitoneal administration could be extrapolated to oral intake of tea beverages [33].

As for caffeic acid, a higher abundance was observed in fermented teas, both black teas and pu-erh (0.57–0.98 mg·g^−1^), albeit associated with a decrease in its concentration (see Fig. 3b). The interaction with digestive enzymes, α-amylase, pepsin and pancreatin, together with its higher reactivity under intestinal pH, seems to account for its lack of detection at the duodenal stage in any of the teas evaluated (Table 3). Similarly, Gayoso et al. [34] reported a poor intestinal bioaccessibility of caffeic acid standard, recovering 8% of the initial compound after a simulated in vitro digestion.

In the case of ferulic acid, a higher stability in non-fermented white and green (normal and decaffeinated) teas than in fermented black teas was observed (see Fig. 3b). Indeed, ferulic acid was greatly bioaccessible after the intestinal phase in all the infusions in which was quantified (IVBA > 45%), except in pu-erh tea due to its low concentration. This result agrees with some studies that have reported high stability of ferulic acid after in vitro pancreatic digestion [34]. In black varieties, a relentless decline from the salivary stage to the intestinal phase was observed, due to the combination of its interaction with digestive enzymes and pH changes. Nevertheless, release of ferulic was also noted during the gastric stage (Fig. 4a and d) owing to the acidic hydrolysis of ferulic derivatives. These results are highly noteworthy since in situ and ex vivo models have suggested that ferulic acid may be absorbed from the stomach, and therefore, may exert more rapidly some of its anti-inflammatory, anti-diabetic or antihypertensive properties observed in vivo [35].

Coumaric acid showed a rather stable digestive behaviour in green and black tea than white tea (Fig. 3b), where a poorer bioaccessibility was found. Bioactive changes triggered by the rolling step carried out in both teas may contribute to improve the stability of this phenolic acid. Like the hydroxycinnamic acids aforementioned, the concentration of the aglycone coumaric acid increased after the acidic hydrolysis in the gastric phase in green and decaffeinated green tea (Fig. 4c and d), showing a IVBA over 150%. A further release was observed in the intestinal phase of green decaffeinated tea, like that described in the bioaccessibility studies on apple varieties [33].

As for the dihydroxybenzoic acids, gallic acid was the main phenolic acid quantified in the digestion process, and nonetheless, approximately less than half of the initial concentration reached the intestine in most of the teas evaluated, mainly after the intestinal phase (see Fig. 3b). Overall, the instability of gallic acid might be ascribed to the three adjacent hydroxyl groups in positions 3, 4 and 5 of the hydroxybenzoic moiety, which makes it more vulnerable to semiquinone free radical formation at mild alkaline pH, when a proton is donated [14]. Still, a significant release was observed in green tea (Fig. 4c) most likely to the degradation of larger phenolic structures, such as epigallocatechin or epigallocatechinagallate, commonly found in this variety [12, 14]. This trend was not observed in decaffeinated green tea (Fig. 4d), probably due to its lower concentration in polymeric catechin derivatives because of the lower prooxidant action of caffeine [10].

Dihydroxybenzoic acid was more stable in yellow, both oolong teas and post-fermented pu-erh tea (see Fig. 3b). It was in semi-fermented oolong teas where an intestinal release up to 0.262 mg·g^−1^, noted in Fig. 4e and f, led to a noticeable bioaccessibility (IVBA 60% and 150%), probably due to the presence of more complex dihydroxybenzoic acid derivatives [29].

Resveratrol stilbene was found in all the varieties other than white tea, which may suggest that resveratrol release occurs when tea leaves undergo a further processing, either by steaming, sweltering, rolling or even by the action of oxidases in the tea matrix. This phenolic compound has been scarcely documented in teas, due to its low abundance, but the research on its bioaccessibility is rather relevant due to its anti-inflammatory, antiproliferative, anti-ageing and cardioprotective role [36]. In this line, resveratrol proved to be highly unstable, with a remarkable degradation in gastric or duodenal environments in the decaffeinated green and oolong varieties and, therefore, showing an IVBA = 0% in all the teas analysed (see Fig. 3b). The low water solubility of resveratrol, together with its sensitivity to environmental oxygen, pH, temperature and digestive enzymes, has been identified as some of the reasons for its low bioaccessibility [36].

#### Tea flavonoids

Regarding flavonoid behaviour, flavan-3-ol catechin was not only more abundant in unfermented teas but also more stable throughout digestion (Fig. 3c). Catechin showed a greater bioaccessibility in white tea than green, despite IVBA values below 50%. Other authors have found that flavon-3-ol bioaccessibility was highest in white tea, followed by green and finally black tea, although lower values were reported [14]. As noted in this study, the main decrease in catechin was observed in the intestinal phase because of the oxidation of its backbone at mild alkaline pH [12].

In the case of myricetin, narangin, hesperidin and kaempferol have been claimed to be minor flavonoids in tea [15], as is evident from the results obtained (Table 2). Neither myricetin nor narangin was detected from the salivary step, while hesperidin reached the duodenal stage in white, yellow and both oolong teas (Fig. 3c). Interestingly, hesperidin and myricetin showed a further release in the intestinal phase of white tea (Fig. 4a), attaining IVBA of 769% and 78%, respectively, feasibly explained by a hydrolysis under mild alkaline pH of high molecular weight glycosides, such as myricetin-3-*O*-rhamnodiglucoside or myricetin-3-*O*-galactoside [6, 33]. However, kaempferol showed a higher digestive stability in the yellow environment, although not bioaccessible at the duodenum (Fig. 4b). Similarly, Rha et al. [11] reported that kaempferol was considerably stable under gastric conditions, albeit it was mostly degraded (95–97%) after being subjected to intestinal treatments in green tea extracts.

The flavonol rutin showed a gradual degradation during the digestive pathway, with the highest stability in unfermented green teas. Decaffeinated green tea appeared to diminish rutin degradation according to its high IVBA of 95%, largely driven by the lack of caffeine, which may enhance the flavonol oxidation at mild pH. As can be seen in Fig. 3c, rutin degradation started at the salivary phase, particularly in the semi-fermented oolong teas. Subsequently, there was a decrease driven by rutin-pepsin interaction via non-covalent bonds, together with the acidic pH of the gastric phase, which favoured the cleavage of the *O*-glycosidic bonds of rhamnose and glucose from the rutin backbone [37, 38]. Nevertheless, a release of rutin was found in the gastric phase of the oolong teas (Fig. 4e and f), caused by the cleavage of other high molecular weight rutosides present in these teas upon their partial fermentation. According to the current finding, Celep et al. [37] described a delay in the effects of gastric medium on rutin degradation compared to the intestinal phase in fruit wines.

Finally, quercetin showed a greater stability in semi-fermented and fermented teas (Fig. 3c), with bioaccessibility rates above 100% in oolong teas. This result is all the more appealing as quercetin has been associated in several works with antioxidant, antidepressant and anticarcinogenic property effects all of which could be displayed by the consumption of oolong teas [3, 33]. Thus, the fermentation stage, in which polyphenol glycosides are formed, played a crucial role for the subsequent release of quercetin during the digestive process. Furthermore, under gastric conditions, an increase in quercetin concentration was observed (Fig. 4f), probably due to the excision of the *O*-glycosidic bond of quercetin derivatives, such as quercetin-3-*O*-α-L-rhamnoside and quercetin-3-*O*-β-D-glucopyranoside, present in oolong teas [9]. Additionally, quercetin is the aglycone of rutin, so after cleavage of the sugar moiety of rutin, an increase in quercetin would be expected (Fig. 4a and d–f). However, no such rise was observed in black or red tea (Fig. 4g–i), which could result from the presence of higher molecular weight derivatives, either because of their higher degree of fermentation or because of the extreme delicacy of quercetin at alkaline pH in the intestinal phase [37].

#### Multivariate analysis of teas: digestion and bioaccessibility

To assess the behaviour of the teas studied during the in vitro digestion process as well as their duodenal bioaccessibility, according to their phenolic and caffeine content, a multivariate CA and a PCA were performed.

**Fig. 5:**
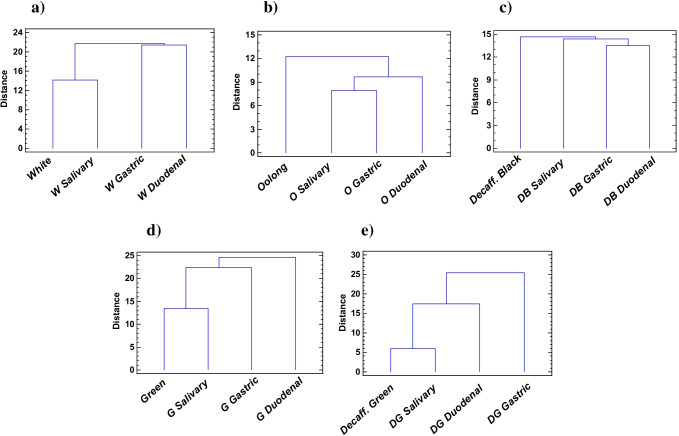
Dendogram of cluster analysis of the tea infusions during the in vitro digestion process: **a** white, **b** oolong, **c** black decaffeinated, **d** green and **e** green decaffeinated tea. Euclidean and nearest-neighbour distances were used to sort tea digestion stages into clusters

Figure 5 shows the dendograms obtained for white, green, decaffeinated green, oolong and decaffeinated black tea. The dendograms of yellow, milk oolong and black tea were quite similar to the one displayed by white tea (Fig. 5a), while pu-erh tea showed a comparable dendogram to black decaffeinated tea (Fig. 5c). As it can be seen, in general, no dissimilarities were found between the salivary phase and the initial infusion for the non-fermented teas (white, yellow and both green ones), together with milk oolong and black tea, as reported somewhere else [14]. Presumably, this is owing to the higher enrichment of non-fermented, milk oolong and black teas in quercetin, kaempferol, gallic acid, ferulic acid and chlorogenic acid, along with some theaflavins and thearugbins in oxidised teas, which have been stated to be effective in reducing α-amylase catalytic activity by both competitive and non-competitive mechanisms [39, 40].

In contrast, to oolong (Fig. 5b) pu-erh and decaffeinated black tea (Fig. 5c), where the digestion of polyphenols was already initiated under oral biochemical conditions, either by a weaker inactivation of α-amylase or by a more noticeable impairment of the mild alkaline pH of saliva, which hinders the stability of phenolic compounds [10]. Thus, again, the decaffeination process seems to have a greater impact on the phenolic composition and digestive behaviour of black tea than green tea; moreover, the microbial post-fermentation process in pu-erh tea seemed to lead to a less stable environment than the oxidative fermentation occurred in black tea. Besides, the differences between oolong tea (Tieguanying) and milk oolong tea (Jin Xuan) were found to play a significant role particularly in the salivary behaviour of flavonoids (as shown Fig. 3c).

Meanwhile, a significant change was observed in the gastric phase for all teas tested, except for oolong tea (Fig. 5b), essentially driven by the preservation of the profile and concentration of the flavonoids detected, whose glycosides have been described as stable at acidic pH [33]. As opposed to phenolic acids which may appear less stable, either because of the acidic gastric environment or because of their interaction with pepsin [10].

The greater distance between the duodenal stage and the initial infusions supported that degradation of the bioactive compounds might occur chiefly in the intestine, where a mild alkaline pH may facilitate epimerisation and auto-oxidation of the phenolic compounds with dissolved residual oxygen [11, 14]. On the other hand, the interaction of phenolic compounds with pancreatin may also decrease the free form of the phenolic compound, although this effect is usually less severe than the one of pH [10]. However, a large difference between gastric and duodenal phase was only found for unfermented green teas and oolong tea, probably due to the unusual release of phenolic acids and flavonoids into the duodenum. This shows that green tea processing and oolong tea harvesting have resulted in less stable duodenal environments, causing the transformation of polyphenolic derivatives (e.g. quercetin glycosides, epigallocatechins or gallocatechins) into the simple monomers evaluated, as reported elsewhere [11, 15].

**Fig. 6:**
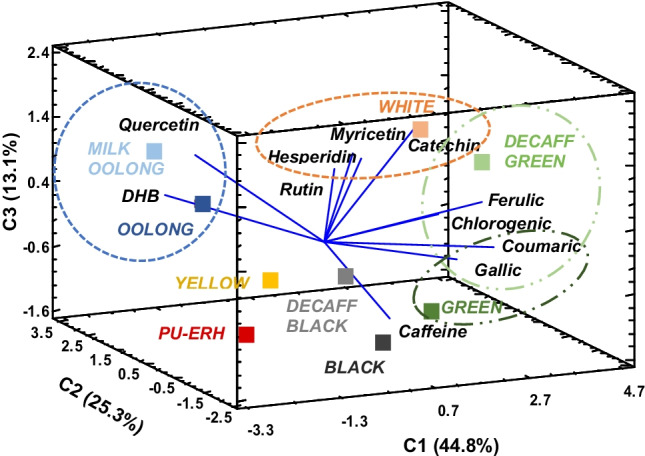
Three-dimensional PCA plot of nine tea infusions (scores, mean values *n* ≥ 6) in relation to the bioaccessibility indices (IVBA) of the polyphenols studied and caffeine. The ellipses show the association of the analytes (loadings) and samples

Finally, Fig. 6 depicts the bioaccessibility pattern of the studied teas by a three-dimensional PCA model, which explained 83.2% of the total variance. The higher fermentation achieved in black tea and pu-erh tea seemed to hinder the potential duodenal availability of the compounds evaluated, as well as for the yellowing process, which has been described as different from that of the other non-fermented varieties. Otherwise, white tea favoured the bioaccessibility of flavonoids such as hesperidin, myricetin and catechin (IVBA 46–769%). Green tea processing ensured high duodenal availability of phenolic acids, namely, coumaric and gallic acid, together with chlorogenic and ferulic acid (IVBA indices above 100%), especially in decaffeinated green tea. In addition, blue processing increased the bioaccessibility of DHB acid and the flavonol quercetin (IVBA 66–180%).

Hence, unfermented white and green teas, as well as oolong teas, achieved a fruitful duodenal availability, above 90%, of some phenolic compounds with reported biological activity (i.e. antidepressant of quercetin or the anti-anxiety of chlorogenic acid) that may be absorbed intact from the small intestine [15, 33], suggesting efficient healthcare effects related to their consumption.

The results obtained support that bioaccessibility is not only dependent on the initial concentration but also on the composition of the matrix, in which the processing of the tea plays a crucial role. Thus, in vitro digestion procedures might be considered a feasible approximation to the physiological conditions of digestion, providing useful information on the role of tea type in the availability of polyphenols with health effects. However, in vivo and clinical studies should be further performed to fully establish and correlate the systematic bioactivities of teas with bioavailability [15].

## Conclusions

The current study has clearly demonstrated how tea type and processing significantly influences the pattern, gastrointestinal stability and bioaccessibility of some tea phenolic compounds and caffeine, and, thus, on their relationship to health benefits.

Gallic acid and rutin are the most abundant phenolic compounds in the raw tea infusions evaluated (13.4 g·mg^−1^ and 63 g·mg^−1^, respectively), where unfermented teas stood out for their greater antioxidant activity (689–441 mg GAE·g^−1^) and total content of individual polyphenols (30–66 mg GAE·g^−1^). Furthermore, the decaffeination process seemed to protect the degradation of the polyphenols evaluated both during processing and in vitro digestion.

After the simulated in vitro digestion, some of the target polyphenols were not found in the duodenum (kaempferol, resveratrol, narangin or caffeic acid), while others resulted remained (rutin) or even gave rise (gallic acid, quercetin or chlorogenic acid) by the cleavage of other high molecular weight polyphenols present in the teas.

On the other hand, the application of both PCA and CA analysis allowed designating white, green and oolong teas as potential candidates for enhancing the bioaccessibility (IVBA > 90%) of bioactive phenolic compounds with antioxidant, antidepressant or the anti-anxiety effects. It also evidenced the importance of multivariate analysis for understanding the polyphenol transformations during the digestion process, as well as the influence of such transformations in the bioaccessibility of tea polyphenols.

Nonetheless, further studies on the interaction of tea polyphenols with the gut microbiome and mucosa are warranted to guide the understanding of the behaviour and physiological effects of polyphenols and caffeine after tea ingestion.

## Supplementary Information

Below is the link to the electronic supplementary material.

Supplementary file1 (DOCX 3181 KB)

## Data Availability

The authors confirm that the data within the article and/or the supplementary material supports the findings of this study.
