# Supplementary material for: A combined analytical-chemometric approach for the in vitro determination of polyphenol bioaccessibility by simulated gastrointestinal digestion
Source: Anal Bioanal Chem. 2022 Feb 2;414(8):2739–55. doi: 10.1007/s00216-022-03922-x (PMC8888401; doi:10.1007/s00216-022-03922-x)
Supplement: Supplementary file 1 — Supplementary file1 (DOCX 3181 KB) [file 216_2022_3922_MOESM1_ESM.docx]

**A combined analytical-chemometric approach for the *in vitro* determination of polyphenol bioaccessibility by simulated gastrointestinal digestion**

Esther Gómez-Mejía*, Noelia Rosales-Conrado, María Eugenia León-González, Alejandro Valverde, Yolanda Madrid

Analytical Chemistry Department. Faculty of Chemistry. Complutense University of Madrid. 28040-Madrid, Spain.

(*) To whom correspondence must be addressed: egomez03@ucm.es

Phone number: +34 91 394 4318

**Appendix - Supplementary Tables**

**Table S1**. Retention time, wavelengths for quantitative UV analysis (λ_max_), and mass spectral data for the identification and quantification of phenolic compounds and caffeine in tea infusions and digested extracts by chromatographic methods.

| **Compound** | **Retention time**  **(min)** | | **Quantification wavelength**  **(nm)** | **Precursor Ion**  **(*m/z*)** | | **MS/MS transitions**  **(*m/z*)**  **(Collision Energy, eV)** |
| --- | --- | --- | --- | --- | --- | --- |
|  | **DAD** | **MS/MS** |  |  |  |  |
| **Gallic acid** | 3.1 | 6.2 | 292 | 169.0 | [M-H]ˉ | *125.1* (19), 79.0 (23), 53.1 (20) |
| **Dihydroxybenzoic acid** | 3.7 | 10.2 | 260 | 153.0 | [M-H]ˉ | *109.0* (16), 108.1 (25), 53.0 (25) |
| **Catechin** | - | 14.4 | - | 289.1 | [M-H]ˉ | *245.2* (15), 109.0 (23), 123.0 (31) |
| **Caffeine** | 7.5 | 15.7 | 260 | 195.0 | [M+H]^+^ | *138.0* (-23), 41.9 (-22), 110.0 (-23) |
| **Chlorogenic acid** | 5.8 | 16.8 | 310 | 353.1 | [M-H]ˉ | *191.0* (16), 85.1 (44), 93.1 (42) |
| **Caffeic acid** | - | 18.1 | - | 179.0 | [M-H]ˉ | *135.0* (18), 134.0 (30), 89.0 (34) |
| ***p*-Coumaric acid** | 12.3 | 23.0 | 310 | 163.0 | [M-H]ˉ | *119.0* (17), 93.0 (31), 117.1 (34) |
| ***trans*-Ferulic acid** | 13.2 | 23.9 | 310 | 193.2 | [M-H]ˉ | *134.0* (18), 178.0 (17), 149.1 (15) |
| **Naringin** | 14.4 | 27.5 | 292 | 579.0 | [M-H]ˉ | *271.2* (38), 151.1 (45), 459.0 (27) |
| **Hesperidin** | - | 27.6 | - | 609.1 | [M-H]ˉ | *301.2* (23), 286.2 (48), 164.0 (55) |
| **Rutin** | 13.8 | 28.5 | 260 | 609.0 | [M-H]ˉ | *300.1* (42), 300.1 (33), 271.0 (55) |
| **Myricetin** | - | 30.3 | - | 317.0 | [M-H]ˉ | *151.0* (26), 179.0 (20), 137.0 (27) |
| **Resveratrol** | 17.5 | 30.7 | 310 | 227.1 | [M-H]ˉ | *143.1* (25), 113.0 (10), 69.1 (21) |
| **Quercetin** | 17.6 | 32.9 | 365 | 301.0 | [M-H]ˉ | *151.0* (22), 179.0 (19), 121.1 (28) |
| **Kaempferol** | 19.7 | 34.9 | 365 | 284.9 | [M-H]ˉ | *93.4* (41), 187.1 (28), 117.1 (43) |

MS/MS *Quantitative transition* is presented in italics.

**Table S2**. Analytical parameters of the cHPLC-DAD method employed for the determination of polyphenols and caffeine

| **Compound** | **Linear range**  **(µg∙L^-1^)** | ***LOD***  **(L∙µg ^-1^)** | ***LOQ***  **(L∙µg ^-1^)** | **Calibration equation, *y = ax + b*** | | | **Intra-day repeatability**  **(n=3) RSD (%)** | | **Inter-day repeatability**  **(N=9) RSD (%)** | |
| --- | --- | --- | --- | --- | --- | --- | --- | --- | --- | --- |
|  |  |  |  | ***a* (L∙µg ^-1^)** | ***b*** | ***R^2^*** | ***k*** | **Area** | ***k*** | **Area** |
| **Gallic acid** | 20-2000 | 2.8 | 9.3 | 3.2 ± 0.1 | 114 ± 47 | 0.9980 | 5.4; 11.3 | 10.4; 10.9 | 13.5; 12.5 | 10.4; 11.2 |
| **Dihydroxybenzoic acid** | 10-75 | 3.0 | 10 | 1.17 ± 0.08 | 136 ± 3 | 0.9833 | 10.3; 5.0 | 21.0; 12.3 | 10.1; 9.0 | 20.7; 8.7 |
| **Catechin** | - | - | - | - | - | - | - | *-* | *-* | *-* |
| **Caffeine** | 90-5000 | 6.0 | 20 | 1.53 ± 0.02 | 0 ± 47 | 0.9978 | 2.3; 3.5 | 2.9; 2.4 | 1.2; 4.0 | 2.8; 3.0 |
| **Chlorogenic acid** | 40-400 | 12 | 39 | 1.37 ± 0.08 | 0 ± 11 | 0.9919 | 3.3; 5.5 | 1.1; 5.7 | 4.9; 6.3 | 4.8; 4.9 |
| **Caffeic acid** | - | - | - | - | - | - | *-* | *-* | *-* | *-* |
| ***p*-Coumaric acid** | 20-400 | 4.9 | 16 | 6.2 ± 0.3 | 0 ± 67 | 0.9842 | 0.9; 3.8 | 1.8; 4.0 | 1.3; 2.7 | 3.1; 5.6 |
| ***trans*-Ferulic acid** | 15-200 | 3.7 | 12 | 4.6 ± 0.1 | 0 ± 13 | 0.9937 | 2.3; 1.9 | 6.6; 3.6 | 2.0; 1.8 | 7.4; 4.4 |
| **Naringin** | 20-60 | 5.1 | 17 | 0.86 ± 0.07 | 19 ± 3 | 0.9849 | 4.1; 0.6 | 2.5; 1.1 | 4.7; 5.9 | 5.2; 6.3 |
| **Hesperidin** | - | - | - | - | - | - | - | *-* | *-* | *-* |
| **Rutin** | 30-4000 | 7.0 | 23 | 0.992 ± 0.007 | 11 ± 10 | 0.9994 | 2.9; 0.6 | 6.8; 5.9 | 2.1; 1.0 | 7.2; 6.1 |
| **Myricetin** | - | - | - | - | - | - | *-* | *-* | *-* | *-* |
| **Resveratrol** | 10-80 | 2.5 | 8.5 | 6.9 ± 0.2 | 4 ± 9 | 0.9940 | 0.3; 0.9 | 1.9; 1.7 | 3.0; 2.2 | 5.7; 4.9 |
| **Quercetin** | 10-80 | 6.3 | 21 | 1.71 ± 0.04 | 0 ± 2 | 0.9952 | 3.0; 0.8 | 2.1; 1.2 | 4.6; 5.1 | 5.9; 4.7 |
| **Kaempferol** | 20-60 | 4.6 | 15 | 1.7 ± 0.3 | 40 ± 10 | 0.9636 | 3.1; 0.7 | 2.2; 1.1 | 9.7; 3.7 | 10.2; 2.6 |

| **Compound** | **Linear range**  **(µg∙L^-1^)** | ***LOD***  **(L∙µg ^-1^)** | ***LOQ***  **(L∙µg ^-1^)** | **Calibration equation, *y = ax + b*** | | | **Intra-day repeatability**  **(n=3) RSD (%)**  **Area** | **Inter-day**  **repeatability**  **(N=9) RSD (%)**  **Area** |
| --- | --- | --- | --- | --- | --- | --- | --- | --- |
|  |  |  |  | ***a* (L∙µg ^-1^)** | ***b*** | ***R^2^*** |  |  |
| **Gallic acid** | 30-130 | 10 | 30 | 700 ± 50 | 0 ± 4657 | 0.9800 | 0.5 | 12 |
| **Dihydroxybenzoic acid** | 10-80 | 3.0 | 10 | 1704 ± 44 | 0 ± 2304 | 0.9973 | 7.7 | 8.7 |
| **Catechin** | 15-130 | 4.5 | 15 | 135 ± 13 | 0 ± 1037 | 0.9749 | 3.4 | 5.4 |
| **Caffeine** | 15-130 | 4.5 | 15 | 7440 ± 217 | 0 ± 17876 | 0.9966 | 5.6 | 5.6 |
| **Chlorogenic acid** | 10-100 | 3.0 | 10 | 2879 ± 11 | 0 ± 715 | 0.9999 | 7.6 | 9.9 |
| **Caffeic acid** | 10-80 | 3.0 | 10 | 3280 ± 80 | 0 ± 4140 | 0.9982 | 8.4 | 14 |
| ***p*-Coumaric acid** | 10-80 | 3.0 | 10 | 1365 ±74 | 0 ± 3828 | 0.9884 | 3.5 | 13 |
| ***trans*-Ferulic acid** | 20-80 | 6.1 | 20 | 64 ± 12 | 1194 ± 534 | 0.9564 | 3.5 | 16 |
| **Naringin** | 20-100 | 6.1 | 20 | 310 ± 10 | 2244 ± 677 | 0.9958 | 5.0 | 11 |
| **Hesperidin** | 10-80 | 3.0 | 10 | 361 ± 35 | 4900 ± 1900 | 0.9818 | 8.8 | 7.3 |
| **Rutin** | 20-100 | 6.1 | 20 | 1030 ± 65 | 0 ± 4297 | 0.9844 | 9.3 | 8.2 |
| **Myricetin** | 5-50 | 1.5 | 5.0 | 1155 ± 64 | 0 ± 2061 | 0.9909 | 3.1 | 6.8 |
| **Resveratrol** | 10-80 | 8.2 | 27 | 42 ± 5 | 0 ± 303 | 0.9644 | 2.7 | 3.4 |
| **Quercetin** | 5-50 | 1.5 | 5.0 | 2127 ± 189 | 0 ± 6576 | 0.9766 | 7.6 | 8.3 |
| **Kaempferol** | 20-80 | 6.1 | 20 | 201 ± 22 | 0 ± 1219 | 0.9518 | 7.7 | 10 |

**Table S3**. Analytical parameters of the HPLC-MS/MS method employed for the determination of polyphenols and caffeine

**Appendix - Supplementary Figures**

**Figure S1**

***Camellia sinensis* fresh leaves**

Drying

***WHITE TEA***

***YELLOW TEA***

Sweltering

Withering

Withering

Drying slowly

Rolling

Withering

Drying

***OOLONG TEA***

Steaming

Withering

Drying

***GREEN TEA***

Rolling/

Shaping

Partial fermentation

Rolling

Withering

Drying

***BLACK TEA***

Full fermentation

***PU-ERH TEA***


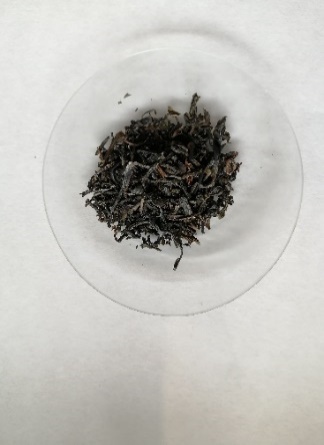

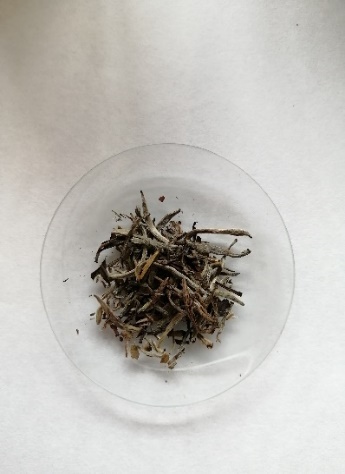

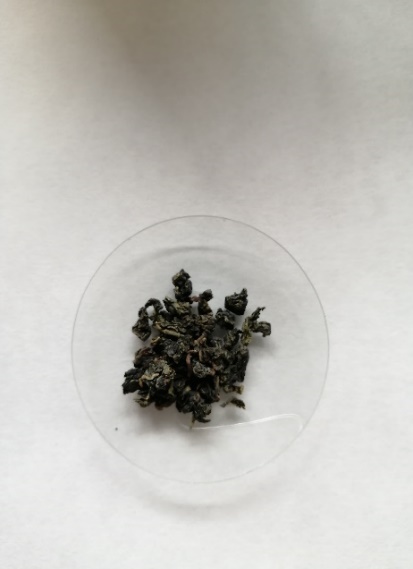

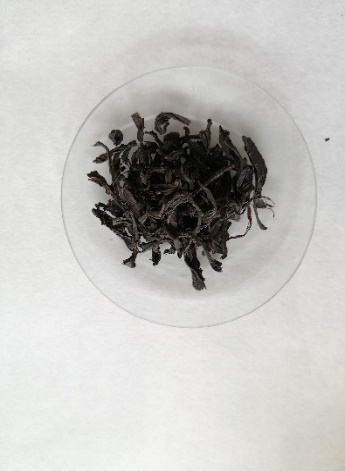

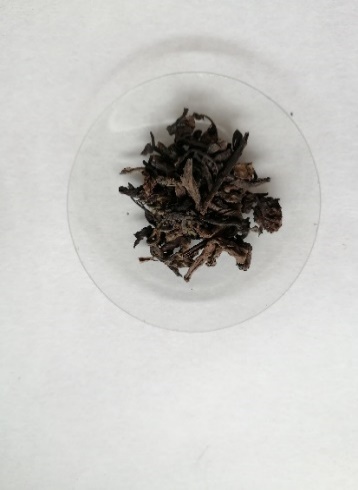

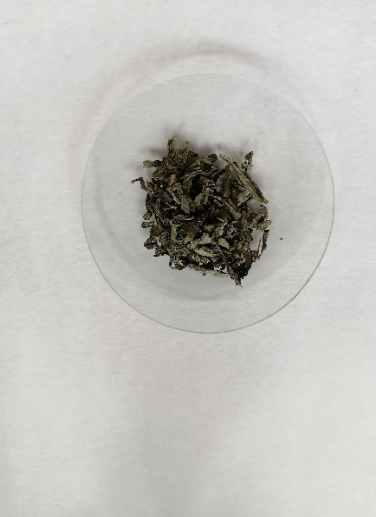


Pan firing

Withering

Steaming and shaping

Sun drying

Rolling

Dry storage

*

*

**Figure S1**. Flow chart of the processing of the different types of tea studied. Key process. *Decaffeination process when applied.

**Figure S2**


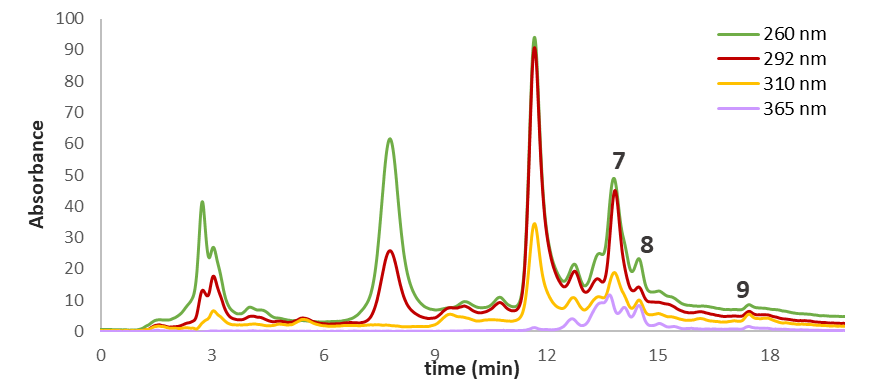


**1**

**2**

**3**

**5**

**6**

**4**

**10***

**a)**

**b)**


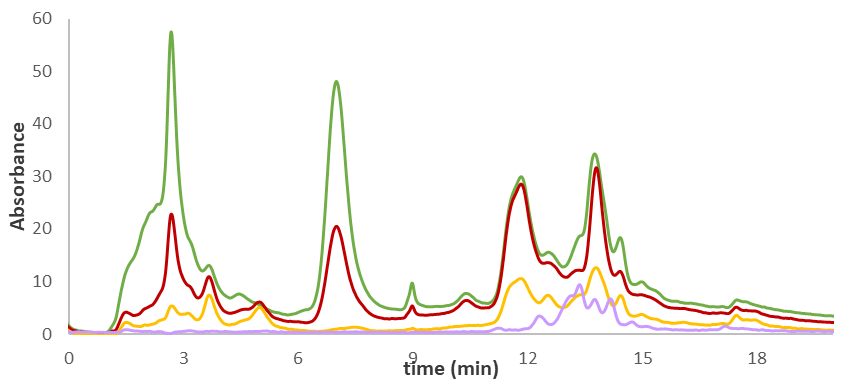


**1**

**2**

**3**

**5**

**6**

**4**

**7**

**9**

**c)**


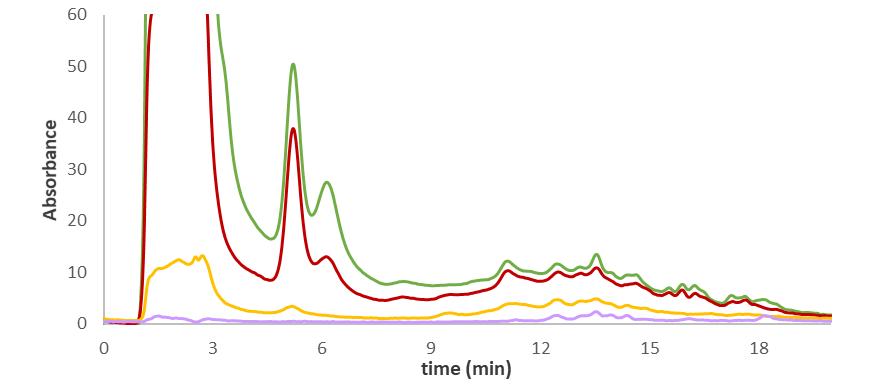


**4**

**5**

**6**

**7**

**1***

**10***

**3***

**d)**


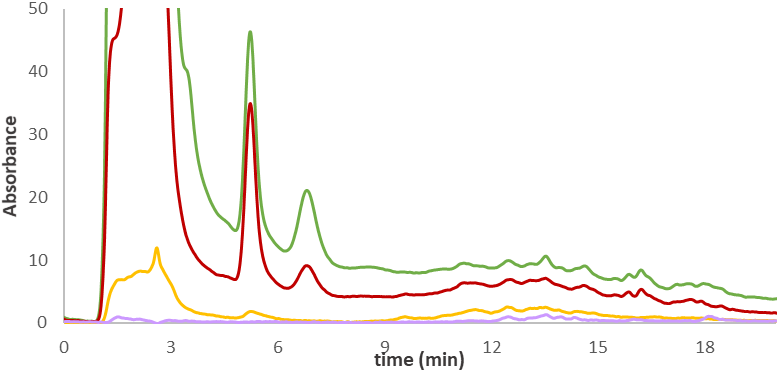


**4**

**5**

**6**

**7**

**1***

**3***

**Figure S2**. HPLC chromatograms of phenolic compounds monitored at 260 nm (green), 292 nm (red), 310 nm (yellow) and 365 nm (violet) in (a) non-digested green tea and (b) salivary, (c) gastric and (d) duodenal phase of digested green tea extracts. Identification of peaks: 1-gallic acid; 2-dihydroxybenzoic acid; 3-chlorogenic acid; 4-caffeine; 5-*p*-coumaric acid; 6-*trans*-ferulic acid; 7-rutin; 8-narangin; 9-resveratrol. *Compounds which were not unequivocally identified nor quantified by HPLC-DAD, being therefore determined by HPLC-MS/MS when possible.

**Figure S3**


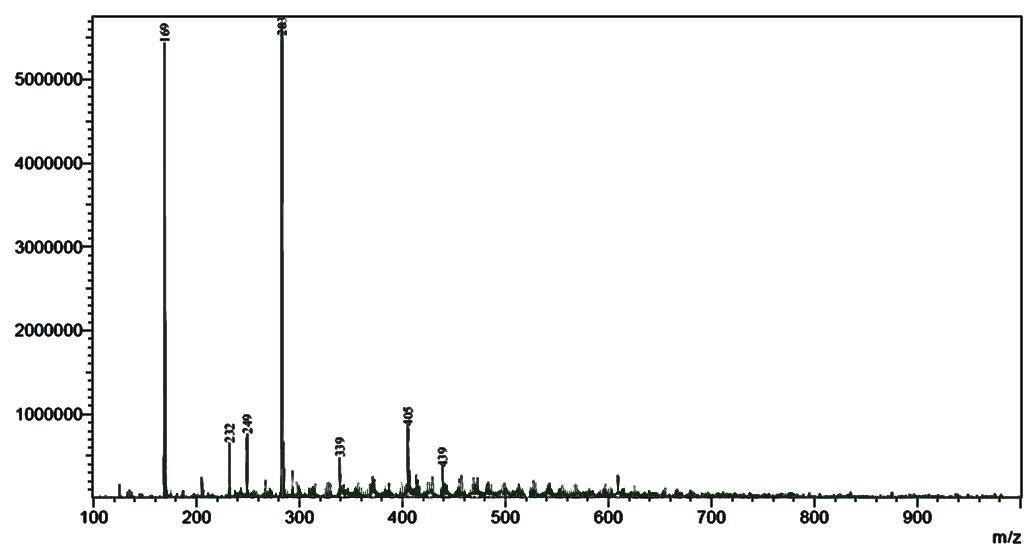


**Gallic acid (169.0 m/z, [M-H]^-^)**

**a)**


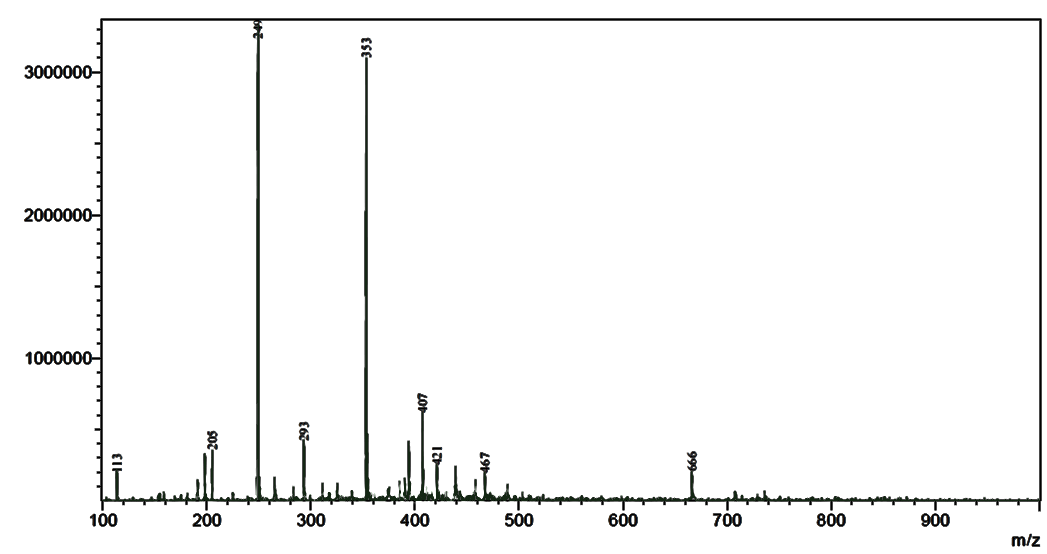


**Chlorogenic (353.1 m/z, [M-H]^-^)**

**b)**


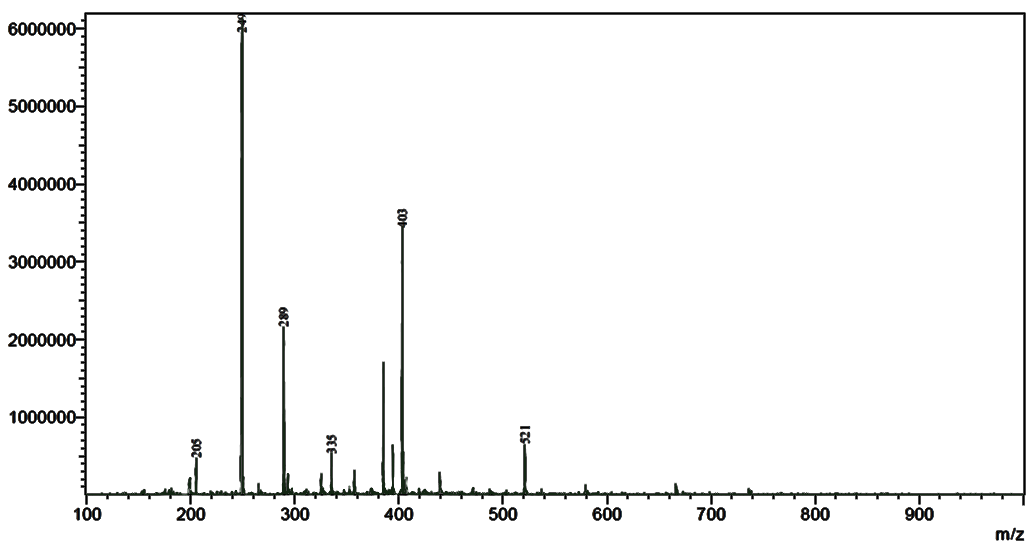


**c)**

**Catechin (289.1 m/z, [M-H]^-^)**


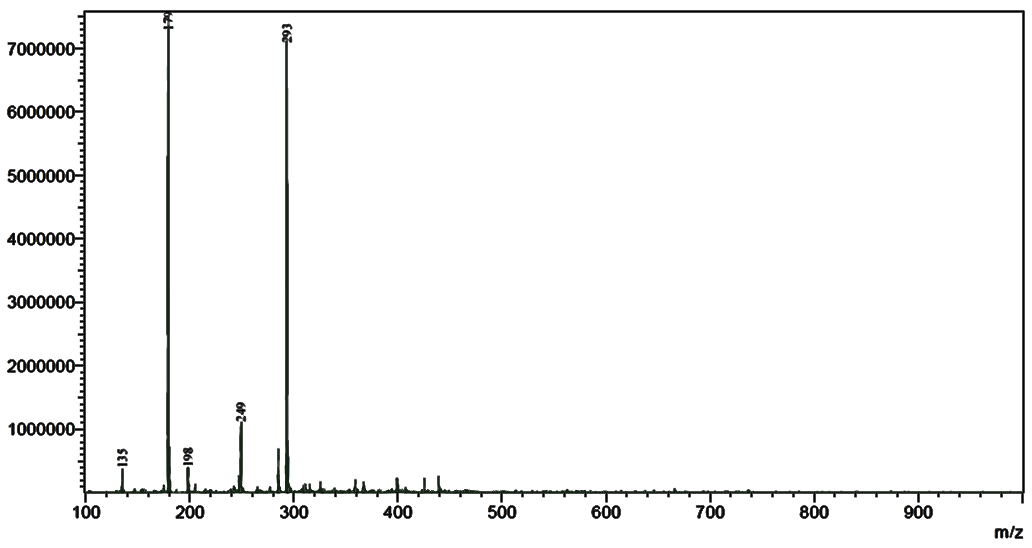


**d)**

**Caffeic (179.0 m/z, [M-H]^-^)**


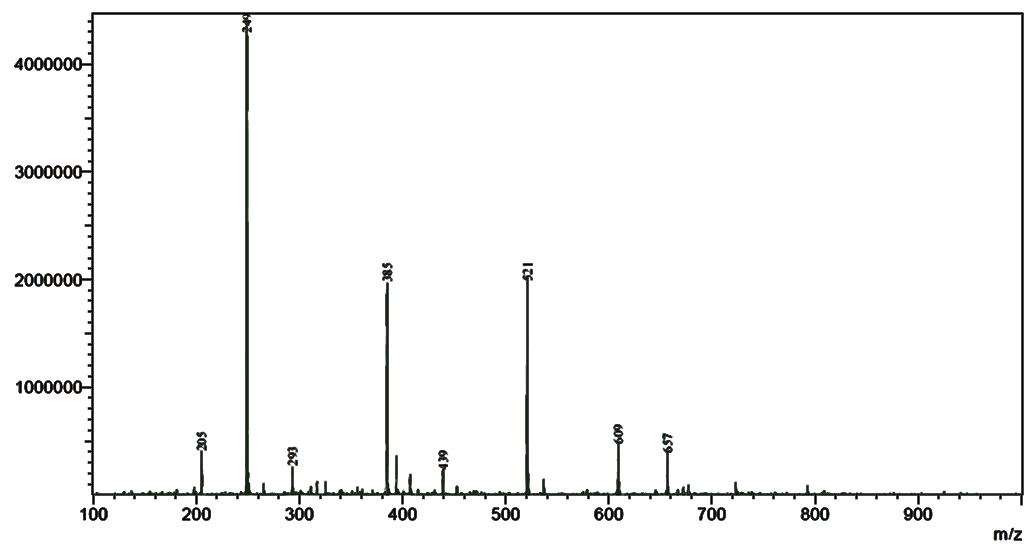


**Hesperidin (609.1 m/z, [M-H]^-^)**

**e)**


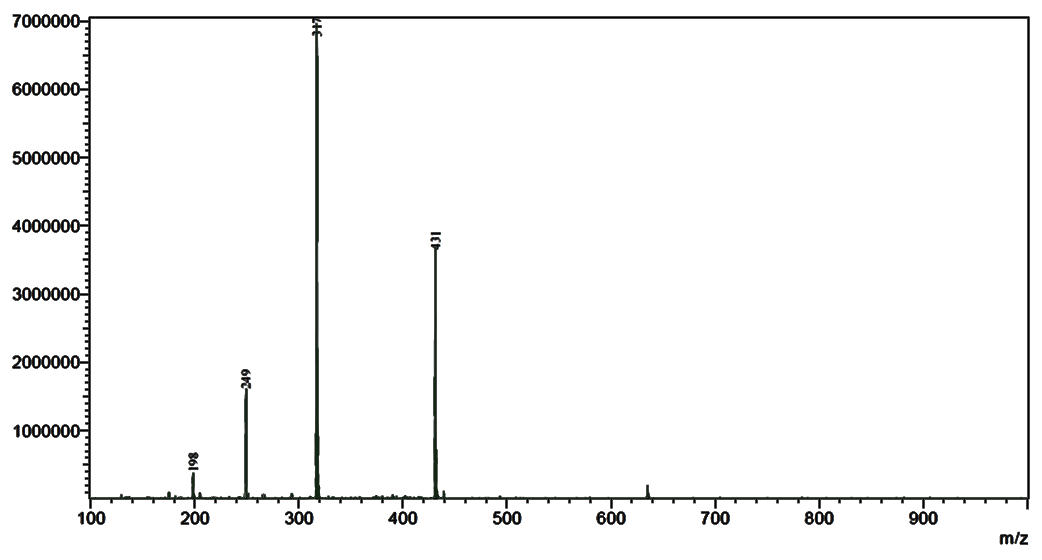


**Myrecetin (317.0 m/z, [M-H]^-^)**

**f)**


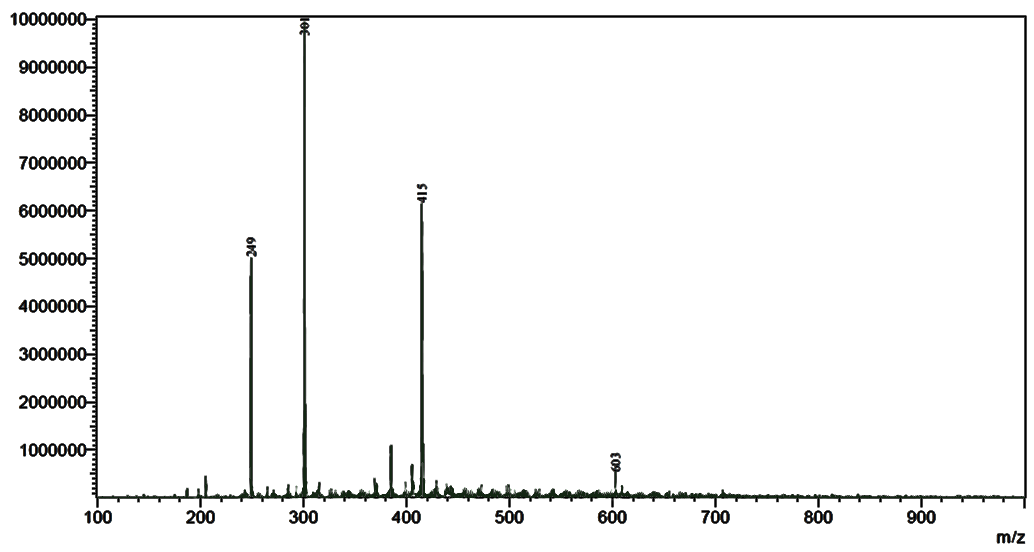


**g)**

**Quercetin (301.0 m/z, [M-H]^-^)**

**Figure S3**: Mass spectra of polyphenols determined by HPLC-MS/MS in non-digested and digested green tea extracts: (a) gallic acid, (b) chlorogenic acid, (c) catechin, (d) caffeic acid, (e) hesperidin, (f) myricetin and (g) quercetin.
